# Ligand-dependent hedgehog signaling maintains an undifferentiated, malignant osteosarcoma phenotype

**DOI:** 10.1038/s41388-023-02864-7

**Published:** 2023-10-16

**Authors:** Vijesh G. Vaghjiani, Catherine R. Cochrane, W. Samantha N. Jayasekara, Wai Chin Chong, Anette Szczepny, Beena Kumar, Luciano G. Martelotto, Andrew McCaw, Kirstyn Carey, Maya Kansara, David M. Thomas, Carl Walkley, Stuart Mudge, Daniel J. Gough, Peter A. Downie, Craig D. Peacock, William Matsui, D. Neil Watkins, Jason E. Cain

**Affiliations:** 1 Hudson Institute of Medical Research, Clayton, VIC 3168 Australia; 2 Department of Molecular and Translational Medicine, School of Medicine, Nursing and Health Sciences, Monash University, Clayton, VIC 3800 Australia; 3 Department of Pathology, Monash Medical Centre, Clayton, VIC 3168 Australia; 4 Peter MacCallum Cancer Centre, Melbourne, VIC 3000 Australia; 5 Sir Peter MacCallum Department of Oncology, The University of Melbourne, Melbourne, VIC 3010 Australia; 6 The Kinghorn Cancer Centre, Garvan Institute of Medical Research, Darlinghurst, NSW 2010 Australia; 7 St.Vincent’s Clinical School, Faculty of Medicine, UNSW, Sydney, NSW 1466 Australia; 8 St. Vincent’s Institute, Fitzroy, VIC 3065 Australia; 9 Department of Medicine, St. Vincent’s Hospital, University of Melbourne, Fitzroy, VIC 3065 Australia; 10 Mayne Pharma International Pty Ltd, Salisbury Sth, SA 5106 Australia; 11 Monash Children’s Cancer Centre, Monash Children’s Hospital, Monash Health, Clayton, VIC 3168 Australia; 12 Department of Paediatrics, Monash University, Clayton, VIC 3168 Australia; 13 Department of Genetics and Genome Sciences, Case Western Reserve University School of Medicine, Case Comprehensive Cancer Center, Cleveland, OH 44106 USA; 14 Department of Oncology and Livestrong Cancer Institutes, Dell Medical School, University of Texas at Austin, Austin, TX 78712 USA; 15 Research Institute in Oncology and Hematology, CancerCare Manitoba, Winnipeg, MB R3E-0V9 Canada; 16 Department of Internal Medicine, Rady Faculty of Heath Sciences, University of Manitoba, Winnipeg, MB R3A-1R9 Canada

**Keywords:** Sarcoma, Bone cancer

## Abstract

*TP53* and *RB1* loss-of-function mutations are common in osteosarcoma. During development, combined loss of TP53 and RB1 function leads to downregulation of autophagy and the aberrant formation of primary cilia, cellular organelles essential for the transmission of canonical Hedgehog (Hh) signaling. Excess cilia formation then leads to hypersensitivity to Hedgehog (Hh) ligand signaling. In mouse and human models, we now show that osteosarcomas with mutations in *TP53* and *RB1* exhibit enhanced ligand-dependent Hh pathway activation through Smoothened (SMO), a transmembrane signaling molecule required for activation of the canonical Hh pathway. This dependence is mediated by hypersensitivity to Hh ligand and is accompanied by impaired autophagy and increased primary cilia formation and expression of Hh ligand in vivo. Using a conditional genetic mouse model of *Trp53* and *Rb1* inactivation in osteoblast progenitors, we further show that deletion of *Smo* converts the highly malignant osteosarcoma phenotype to benign, well differentiated bone tumors. Conversely, conditional overexpression of SHH ligand, or a gain-of-function SMO mutant in committed osteoblast progenitors during development blocks terminal bone differentiation. Finally, we demonstrate that the SMO antagonist sonidegib (LDE225) induces growth arrest and terminal differentiation in vivo in osteosarcomas that express primary cilia and Hh ligand combined with mutations in TP53. These results provide a mechanistic framework for aberrant Hh signaling in osteosarcoma based on defining mutations in the tumor suppressor, TP53.

## Introduction

Hedgehog (Hh) signaling is an evolutionarily conserved pathway required for cell specification and patterning during development [1]. In mammals, canonical hedgehog (Hh) signaling pathway activation is driven by three ligands, sonic hedgehog (SHH), indian hedgehog (IHH), and desert hedgehog (DHH) that are expressed during development in a tissue specific manner, with SHH being the most predominant. In the absence of Hh ligand, the 12-pass transmembrane receptor patched1 (PTCH1) inhibits the G protein-coupled-like receptor smoothened (SMO). In this state, full length GLI proteins are constitutively processed into their transcriptional repressor forms. Binding of ligand to PTCH1 triggers internalization and targeted lysosomal degradation relieving suppression of SMO that then translocates to the primary cilia, a single, immotile membrane-bound organelle that coordinates Hh signal transduction by trafficking of key proteins along a microtubule core. Active SMO prevents processing of full length GLI proteins that transcriptionally activate Hh target genes, including *GLI1* [1, 2]. This cascade of events defines the canonical Hh pathway. Constitutive ligand-independent Hh pathway activation driven by inactivating mutations in *PTCH1* and oncogenic mutations in *SMO* are described in small number of human malignancies, including SHH-driven medulloblastoma and basal cell carcinoma, and can be effectively treated using small molecule SMO inhibitors [3]. However, the vast majority of cancers in which Hh pathway activation is implicated, do not harbor mutations in Hh signaling components and are thought to depend on paracrine and/or autocrine ligand-dependent Hh signaling.

Osteosarcoma is the most common primary tumor of the bone with the highest incidence in children, adolescent and young adults between 10 and 30 years of age [4]. The use of neoadjuvant chemotherapy, surgical resection and adjuvant chemotherapy has significantly improved the 5-year survival of localized osteosarcoma to approximately 70%. However, outcomes for patients with metastatic disease or following disease recurrence remain poor, with a 5-year survival of approximately 30%.

The increased prevalence of osteosarcoma in Li-Fraumeni syndrome [5] and hereditary Retinoblastoma [6], as well as recurrent somatic mutations in *TP53* and *RB1* [7, 8], strongly implicates these tumor suppressor genes in the pathogenesis of the disease. Furthermore, expression of primitive osteoblast markers [9] and dysregulated developmental signaling pathways required for normal bone development are features of osteosarcoma and contribute to its immature phenotype. The Hh signaling pathway is required for normal bone development [10, 11] and aberrant activation of the pathway is associated with osteosarcoma [12–17]. Moreover, we have recently described a role for *Trp53* and *Rb1* in the Hh pathway during development by showing that loss of both genes led to downregulation of autophagy, aberrant formation of primary cilia and hypersensitivity to Hh ligand signaling [18]. In the present study, we sought to investigate the relationship between TP53, RB1 and Hh signaling in osteosarcoma using genetically defined models and elucidate the contribution of dysregulated Hh signaling to the immature osteosarcoma phenotype.

## Results

### Cilia formation and ligand-dependent Hh signaling in murine osteosarcoma

**Fig. 1:**
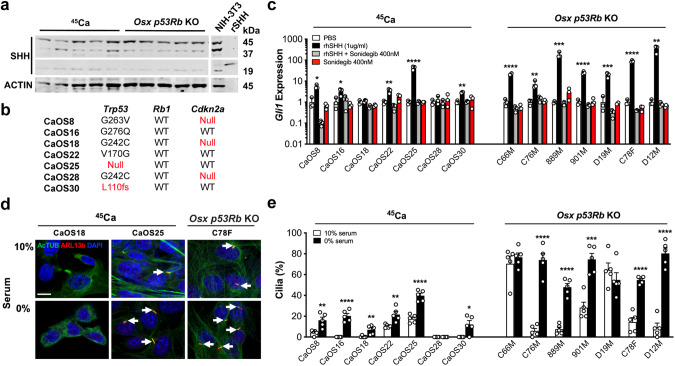
Hedgehog responsiveness and primary cilia formation in mouse osteosarcoma cells. **a** Western blot analysis of SHH and ACTIN expression in radiation induced (^45^Ca) and genetic (*Osx p53Rb* KO) mouse osteosarcoma cell lines. Lysates from NIH-3T3 cells and recombinant SHH (rSHH) are included as positive controls. *N* = 5 independent cell lines per genotype. **b** Mutational status of *Trp53*, *Rb1* and *Cdkn2a* in the ^45^Ca mouse osteosarcoma (CaOS) cell lines. **c** *Gli1* mRNA expression in ^45^Ca and *p53Rb* KO mouse osteosarcoma cell lines treated with PBS vehicle control, 1 μg/ml rhSHH, 1 μg/ml rhSHH and 400 nM sonidegib (LDE225) or 400 nM sonidegib for 24 h. Values are normalized to the expression of *β2-microglobulin. n* = 3, mean ± SEM, *P < 0.05, **P < 0.01, ***P < 0.001, ****P < *0.0001*, one-way ANOVA/Tukey’s test. **d** Representative images demonstrating immunofluorescence colocalization of acetylated α-tubulin (AcTUB) and ARL13B in ^45^Ca (CaOS18, CaOS25) and *p53Rb* KO (C78F) mouse osteosarcoma cell lines cultured in 10% serum, or serum-free media for 24 h. Primary cilia are highlighted by arrows (scale bar, 5 µm). **e** Primary cilia frequency in ^45^Ca and *Osx p53Rb* KO mouse osteosarcoma cell lines cultured in 10% serum or serum-free media for 24 h. *n* = 5 individual replicates per cell line, mean ± SEM, *P < 0.05, **P < 0.01, ***P < 0.001, ****P ≤ *0.0001*, Student’s unpaired *t* test.

Since mutations in both the *TP53* and *RB1* pathways occur frequently in osteosarcoma [19], we explored the relationship between TP53, RB1 and Hh signaling in osteosarcoma using two distinct mouse models: (i) the Ca45 radiation-induced model (^45^Ca) [20]; and (ii) a *Trp53* and *Rb1* conditional genetic model in which lox-P alleles of *Trp53* and *Rb1* are specifically inactivated in the early osteoblast lineage by Cre recombinase under the control of an *Osterix* promoter transgene (here after *Osx p53Rb* KO mice) [21]. Cell lines derived from both models express unprocessed 45-kDa SHH protein and low levels of the 19-kDa active amino-terminal peptide (Fig. 1a, Supplementary Fig. 1a). Furthermore, expression of *Ihh*, *Dhh* and *Shh* mRNA was detected at variable levels across a panel of ^45^Ca and *Osx p53Rb* KO cell lines (Supplementary Fig. 1b, c). ^45^Ca cell lines were characterized by a spectrum of previously undescribed *Trp53* mutations with unknown functional significance, wildtype *Rb1*, and occasional loss of *Cdkn2a* (Fig. 1b). To determine Hh ligand responsiveness, we treated a panel of ^45^Ca and Osx *p53Rb* KO cell lines with SHH ligand. Canonical Hh pathway activation measured by *Gli1* expression in ^45^Ca cell lines was variable (Fig. 1c). In contrast, all *Osx p53Rb* KO cell lines were highly responsive (Fig. 1c). As expected, inhibition of SMO signaling by sonidegib (LDE225) completely abolished exogenous ligand mediated Hh pathway activation in all ^45^Ca and *Osx p53Rb* KO cell lines but had no effect on its own (Fig. 1c). These results suggest that Hh pathway activation in osteosarcoma is primarily via reverse paracrine rather than autocrine stimulation. Consistent with our published data in MEFs [18], primary cilia frequency in murine OS cell lines correlated to Hh responsiveness with non-responsive ^45^Ca cell lines exhibiting few or a complete absence of cilia, even after serum starvation, whilst all *Osx p53Rb* KO osteosarcoma cell lines were highly ciliated under serum free conditions and also displayed abundant cilia in normal serum conditions (Fig. 1d, e).

Notably, the *Cdkn2a* null ^45^Ca cell lines, CaOS18 and CaOS28, were poorly ciliated and non-responsive to SHH ligand. The expression of E2F1 transcriptional targets, *Cdk1*, *Ccne1* and *Cdc6* were similar in *Cdkn2a* null CaOS18 and *Cdkn2a* WT CaOS22 suggesting E2F1-mediated cell cycle regulation is unaltered (Supplementary Fig. 1d). Furthermore, genetic inactivation of *Cdkn2a* in the CaOS30 ^45^Ca cell line, did not result in enhanced response to SHH ligand or primary cilia frequency (Supplementary Fig. 2a–d). To further interrogate the RB pathway, we genetically inactivated *Rb1* in the non-responsive and moderately responsive CaOS28 and CaOS30 ^45^Ca cell lines, respectively (Supplementary Fig. 2e). Similar to *Cdkn2a*, *Rb1* inactivation did not have any effect on *Gli1* mRNA response to SHH ligand or primary cilia frequency compared to the isogenic controls with WT *Rb1* (Supplementary Fig. f–i). This suggests that, at least in the ^45^Ca cell lines, perturbed RB signaling is not required for increased ciliogenesis and response to Hh ligand. In contrast, stable re-expression of P53, RB1 or P53 and RB1 in a highly responsive *Osx p53Rb* KO osteosarcoma cell line (D12M) significantly reduced *Gli1* mRNA in response to SHH ligand treatment and primary cilia frequency, supporting a direct connection between P53, RB1 and Hh pathway responsiveness and suggests that either *Trp53* or *Rb1* inactivation is sufficient for increased response to Hh ligand in this context (Supplementary Fig. 3).

**Fig. 2:**
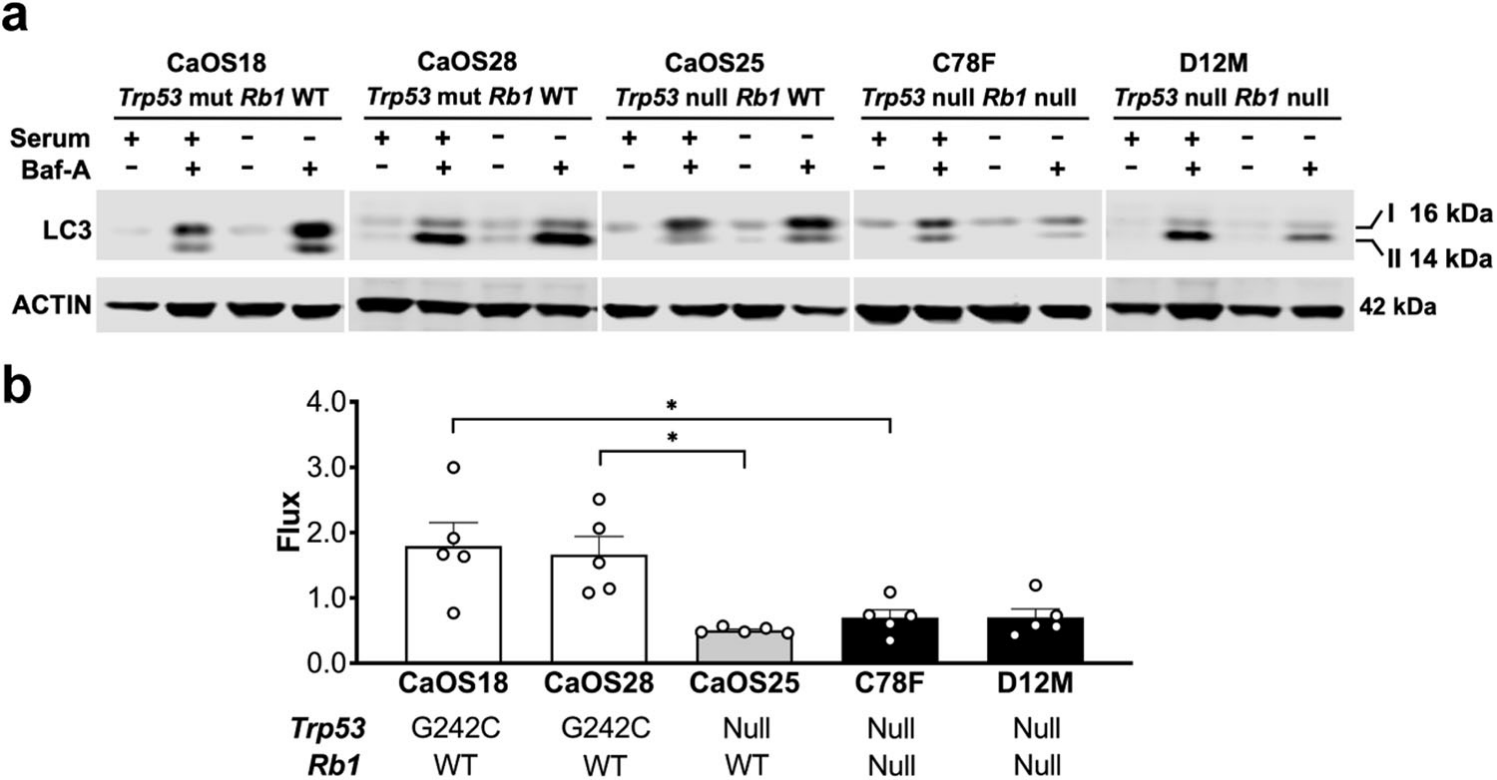
Autophagic flux in ^45^Ca and *p53Rb* KO mouse osteosarcoma cell lines. **a** Western blot analysis of LC3 and ACTIN expression in Hh non-responsive, *Trp53* mutant and *Rb1* WT CaOS18, CaOS28, and Hh responsive, *Trp53* null and *Rb1* WT CaOS25 and *Trp53* null and *Rb1* null C78F, D12M cell lines cultured in 10% serum or serum free media with or without 50 nM bafilomycin-A (Baf-A) for 24 h. *n* = 5 independent experiments. **b** Quantification of autophagic flux by western blot in mouse osteosarcoma cell lines as shown in (**a**). *n* = 5 independent experiments, mean ± SEM. Graph represents fold change in autophagic flux in serum-free media (induced autophagy) compared to normal serum (basal autophagy) *P < 0.05, one-way ANOVA/Bonferroni correction.

The process of ciliogenesis is dynamically regulated during progression of the cell cycle, where cilia are present at G0/G1 stage and are reabsorbed at the mitotic stage [22]. The reabsorption of cilia is facilitated by autophagic recycling of ciliary proteins and defects in autophagy results in dysregulated ciliogenesis [23]. To evaluate whether increased number of cilia is associated with defects in autophagy we examined autophagic flux in selected mOS cell lines by measuring changes in LC3, a marker of autophagosome formation, in the presence of an inhibitor of lysosomal acidification, bafilomycin-A1 (Baf-A), or the lysosomal inhibitor, chloroquine (CHQ) [18, 23]. Analysis of autophagy in mOS cell lines correlated to Hh response with an upregulation of autophagic flux ratio observed in non-responsive ^45^Ca cell lines whilst responsive ^45^Ca and *Osx p53Rb* KO cell lines exhibited a diminished or unchanged autophagic flux ratio consistent with defective inducible autophagy (Fig. 2a, b, Supplementary Fig. 4).

To examine a direct connection of autophagy and ciliogenesis we ablated ATG5, a protein required for autophagosome formation, in the non-responsive ^45^Ca cell line, CaOS18. Unexpectedly, knockdown or knockout of *Atg5* did not lead to an expected increase in ciliogenesis and response to SHH ligand (Supplementary Fig. 5a, b). These results from the murine ^45^Ca osteosarcoma model are in contrast with our previously reported data in mouse embryonic fibroblasts and small cell lung cancer (SCLC) models [18], that implicate TP53 and RB1 in the regulation of Hh responsiveness via autophagy-mediated ciliogenesis.

### Smoothened is required for the osteosarcoma phenotype in *Osx p53Rb* KO mice

**Fig. 3:**
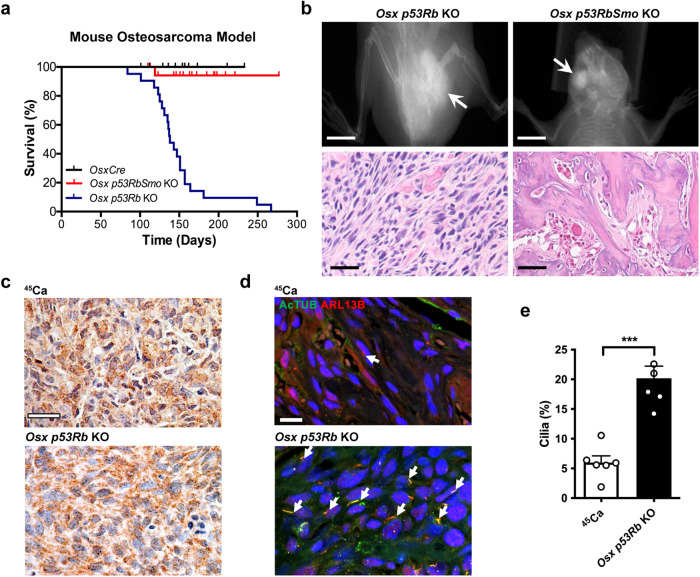
Hedgehog signaling and primary cilia formation in mouse osteosarcoma in vivo. **a** Kaplan–Meier analysis of survival to ethical osteosarcoma endpoint in *OsxCre* (*n* = 29), *OsxCre;Trp53*^*lox/lox*^*;Rb1*^*lox/lox*^ (*Osx p53Rb* KO; *n* = 21), *OsxCre;p53*^*lox/lox*^*;*Rb1^*lox/lox*^*;*Smo^*lox/lox*^ (*Osx p53RbSmo* KO, *n* = 19). *P* < 0.0001, Log-rank (Mantel-Cox) test. **b** X-ray images of a femoral osteosarcoma (arrow) in an *Osx p53Rb* KO and mandibular osteoid osteoma (arrow) in an *Osx p53RbSmo* KO mouse. Scale bar = 1 cm. Corresponding hemotoxylin and eosin stained sections are shown below. Scale bar = 30 µm. **c** SHH expression detected by immunohistochemistry in tumor sections from primary ^45^Ca and *Osx p53Rb* KO mouse osteosarcomas. Immunoperoxidase signal is shown in brown, counterstained with hematoxylin. Scale bar = 40 µm. **d** Immunofluorescence colocalization of acetylated α-tubulin (AcTUB) and ARL13b in primary ^45^Ca and *Osx p53Rb* KO mouse osteosarcoma tumor sections. Scale bar = 15 µm. **e** Primary cilia frequency in ^45^Ca and *Osx p53Rb* KO mouse osteosarcoma. *n* = 6 indepe*n*dent tumors, mean + SEM, *** *P* < 0.001, Student’s unpaired *t* test.

Since canonical Hh signaling through SMO is both ligand and cilia-dependent, we determined whether conditional deletion of *Smo* in the *Osx p53Rb* KO model would affect the penetrance of the osteosarcoma phenotype. To inactivate Hh signaling in this model, we bred these animals with mice carrying a conditional *lox-P* null *Smo* allele. Recombination of alleles in tumor tissue was confirmed by genomic PCR (Supplementary Fig. 6). Genetic inactivation of *Smo* on the *OsxCre p53Rb* KO background (*Osx p53RbSmo* KO) almost completely prevented development of osteosarcoma (Fig. 3a). Strikingly, all *Osx p53RbSmo* KO mice developed small, calcified masses, predominantly on the mandible (Fig. 3b). Pathological assessment revealed features consistent with osteoid osteoma, a benign tumor of the bone (Fig. 3b). Notably, *Osx p53RbSmo* KO tissue exhibited reduced expression of *Smo* and Hh target genes consistent with *Smo* inactivation and loss of Hh pathway activity, and increased expression of terminal osteoblast markers consistent with the observed calcification (Supplementary Fig. 7a, b). Furthermore, sustained pharmacological Hh pathway inhibition in *Osx p53Rb* KO cells with sonidegib in vitro, resulted in increased alizarin red staining, a marker of calcium deposition, reduced expression of *Gli1* and increased expression of terminal osteoblast markers (Supplementary Fig 7c, d). This result suggests that although the loss of two potent tumor suppressors is sufficient to initiate tumor formation, Hh signaling is required to maintain an undifferentiated state and for complete penetrance of a malignant phenotype.

In keeping with our in vitro data, primary *Osx p53Rb KO* tumors and *Osx p53RbSmo KO* osteomas express SHH and display prominent primary cilia (Fig. 3c–e, Supplementary Fig. 7e–g), consistent with TP53 and RB1 inhibiting ligand responsiveness and primary cilia frequency. In contrast, even though primary ^45^Ca tumors do express SHH they exhibit few if any observable primary cilia (Fig. 3c–e). These data strongly support a requirement for Hh signaling in a developmentally regulated tumor model driven by combined loss of TP53 and RB1 that expresses both primary cilia and HH ligand in vivo.

### Aberrant cilia formation, autophagy and Hh pathway expression in human osteosarcoma

**Fig. 4:**
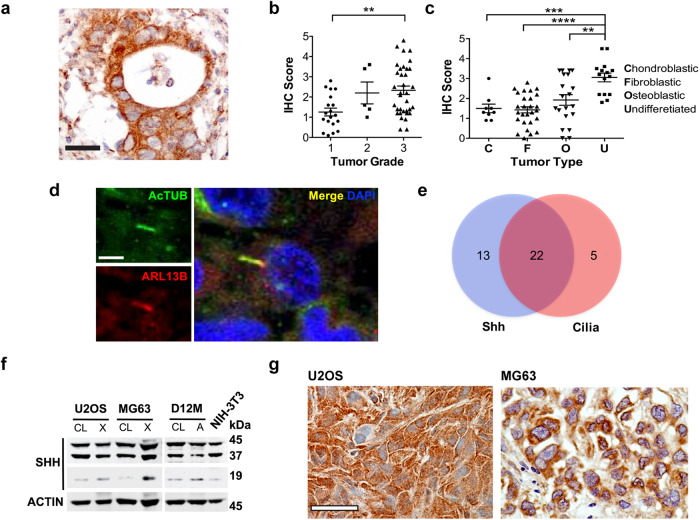
Hedgehog signaling and primary cilia formation in human osteosarcoma in vivo. **a** SHH expression detected by immunohistochemistry in tumor sections of human osteosarcoma. Immunoperoxidase signal is shown in brown, counterstained with hematoxylin. Representative image is shown. Scale bar = 40 µm. **b** Quantification of SHH expression in human osteosarcoma tissue detected by immunohistochemical staining according to tumor grade. ** *P* < 0.01, one-way ANOVA/Tukey correction. **c** Quantification of SHH expression in human osteosarcoma tissue detected by immunohistochemical staining according to tumor subtype. **P < 0.005, ***P < 0.001, ****P < 0.0001, one-way ANOVA/Tukey correction. **d** Immunofluorescence colocalization of acetylated α-tubulin (AcTUB) and ARL13B human osteosarcoma tissue. Scale bar = 5 µm. **e** Relationship between SHH and the presence of primary cilia in human osteosarcoma tissue. *N* = 40. **f** Western blot analysis of SHH and ACTIN expression in human (U2OS, MG63) and mouse (D12M; *Osx p53Rb* KO) osteosarcoma cell lines (CL) and matched xenograft (X) and allograft (A) tissue. Lysate from NIH-3T3 cells is shown as a positive control. **g** SHH expression detected by immunohistochemistry in human osteosarcoma xenografts. Immunoperoxidase signal is shown in brown, counterstained with hematoxylin. Scale bar = 50 µm.

We next investigated the potential importance of Hh signaling in human osteosarcoma. Immunohistochemical staining of Hh ligand using two independent SHH antibodies on 120 human osteosarcoma samples revealed positive staining in 47% of cases and was significantly enriched in high tumor grade and the undifferentiated subtype (Fig. 4a–c; Supplementary Fig. 8a, b). Using an independent cohort of 40 human osteosarcoma samples, we assessed both SHH expression and primary cilia. Here, SHH staining was observed in 88% (35/40) of samples and primary cilia detected in 68% (27/40), with 55% expressing both SHH and primary cilia (Fig. 4d, e). These data are in keeping with previous observations that Hh signaling pathway components IHH, PTCH1, SMO, GLI1 and GLI2 are expressed in human osteosarcoma, and that IHH and GLI2 overexpression is associated with poor patient outcomes [13, 14, 16, 24].

Analysis of SHH expression in human osteosarcoma cell lines, U2OS and MG63 by Western blot showed that while active SHH amino-terminal peptide was expressed at low levels in whole cell lysates, expression was markedly upregulated in xenograft tumor lysates, and in lysates obtained from the *Osx p53Rb* KO cell line D12M allograft tissues (Fig. 4f). This was also confirmed by immunohistochemistry (Fig. 4g; Supplementary Fig. 8c) and suggests that the availability of SHH signaling peptide is greatly enhanced in vivo, thus rendering the tumor cells competent to receive a reverse paracrine signal.

**Fig. 5:**
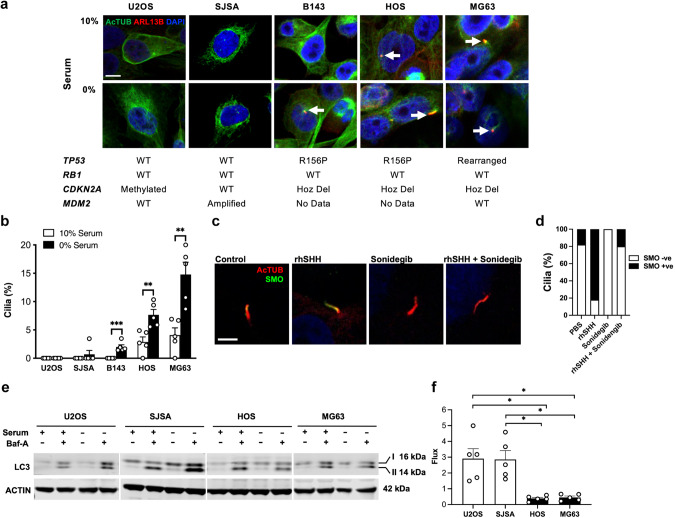
Primary cilia formation and autophagic flux in human osteosarcoma in vitro. **a** Immunofluorescence colocalization of acetylated α-tubulin (AcTUB) and ARL13B in human osteosarcoma cell lines cultured in 10% serum or serum-free media for 24 h. Representative images are shown, along with corresponding genotypes for each cell line. Scale bar = 5 µm. **b** Quantification of primary cilia frequency in human osteosarcoma cell lines cultured in 10% serum or serum-free media for 24 h. *n* = 5 individual experiments, mean + SEM, **P < 0.01, ***P < 0.001, Student’s unpaired *t* test. **c** Confocal immunofluorescence images of primary cilia stained for AcTUB and SMO in B143 human osteosarcoma cells treated with PBS, SHH (rhSHH) and/or sonidegib (LDE225). Scale bar = 5 µm. **d** Quantification of primary cilia co-expressing SMO in B143 human osteosarcoma cells treated with treated with PBS, SHH (rhSHH) and/or sonidegib, *n* = 11–14 ciliated cells per treatement. **e** Western blot analysis of LC3 and ACTIN expression in non-ciliated (U2OS) and ciliated (MG63) human osteosarcoma cell lines cultured in 10% serum or serum-free with or without 50 nM bafilomycin-A (Baf-A) for 24 h. *n* = 5 individual cell lines. **f** Quantification of autophagic flux by western blot in non-ciliated (U2OS) and ciliated (MG63) human osteosarcoma cell lines as shown in (**e**). *n* = 5 independent experiments, mean ± SEM. Graph represents fold change in autophagic flux in serum-free media (induced autophagy) compared to normal serum (basal autophagy) *P < 0.05, Student’s unpaired *t* test.

Using a panel of human osteosarcoma cell lines with previously reported genotypes, we then assessed primary cilia frequency using immunofluorescence. Cilia were either undetectable or at low frequency in human osteosarcoma cell lines, U20S and SJSA, both with wild type *TP53* and *RB1* status (Fig. 5a, b). In contrast, primary cilia were frequently detected in cell lines with reported *TP53* mutations (B143, HOS, MG63) under both serum-starved and normal serum conditions (Fig. 5a, b). Variable expression of *IHH*, *DHH* and *SHH* mRNA was detected in all human osteosarcoma cell lines (Supplementary Fig. 9a, b). Consistent with the mouse ^45^Ca osteosarcoma cell lines, cilia frequency and expression of *CDK1*, *CCNE1* and *CDC6* did not correlate with *CDKN2A* deletion (Supplementary Fig. 9c). To assess response to Hh ligand, we examined Hh target gene expression [25] in an RNA-seq dataset of U2OS and MG63 treated with 1 µg/ml rhSHH for 24 h. In these conditions, we observed no change in *GLI1, HHIP, BCL2, FOXL1* and *PRDM1* expression in U2OS cells (Supplementary Fig. 9d). In contrast, a subtle but nonsignificant increase in *GLI1, HHIP, BCL2, FOXL1* and *PRDM1* was detected in MG63 cells (Supplementary Fig. 9e). Validation of *GLI1* mRNA by quantitative real-time PCR across all cell lines showed a small but significant increase in MG63 cells following ligand treatment that was completely ablated by sonidegib, but no change in HOS, B143, SJSA and U2OS cells (Supplementary Fig. 9f). Furthermore, expression of the Hh pathway target genes *PTCH1* and *HHIP* mRNA was variable and not significantly different between cell lines (Supplementary Fig. 9f) possibly due to limited sensitivity of the analysis in cultures with low primary cilia frequency. As an alternative measure of canonical Hh pathway activation, we visualized the localization of SMO to the primary cilia, a necessary event required for signal transduction [26] in B143 cells. Under serum-starved culture conditions SMO infrequently (<20%) co-localized to the primary cilia and when it did was in low abundance consistent with low levels of Hh signaling activation in these cells (Fig. 5c, d). In response to treatment with SHH ligand, we observed SMO translocation into the primary cilia at a frequency of >80% and with high abundance, mostly co-localizing to 50–80% of the primary cilia length, indicating that these cells are indeed responsive to Hh ligand stimulation. In contrast, basal and ligand-stimulated SMO translocation to the primary cilia was completely abolished or reduced to control levels by treatment with the SMO inhibitor sonidegib (Fig. 5c, d). These data show that Hh signaling is a feature of human osteosarcoma.

To determine if defective autophagy is a feature of ciliated human osteosarcoma cell lines with perturbations in TP53 signaling, we assessed the state of autophagic flux. As expected, the non-ciliated U2OS and poorly ciliated SJSA cell line demonstrated a marked upregulation of autophagic flux in serum free conditions in the presence of Baf-A or CHQ (Fig. 5e, f, Supplementary Fig. 9g, h). In contrast, the highly ciliated MG63 and HOS cell lines failed to upregulate autophagic flux following serum starvation in presence of Baf-A or CHQ (Fig. 5e, f, Supplementary Fig. 9g, h).

### Sustained Hh signaling during osteoblast development blocks terminal differentiation

**Fig. 6:**
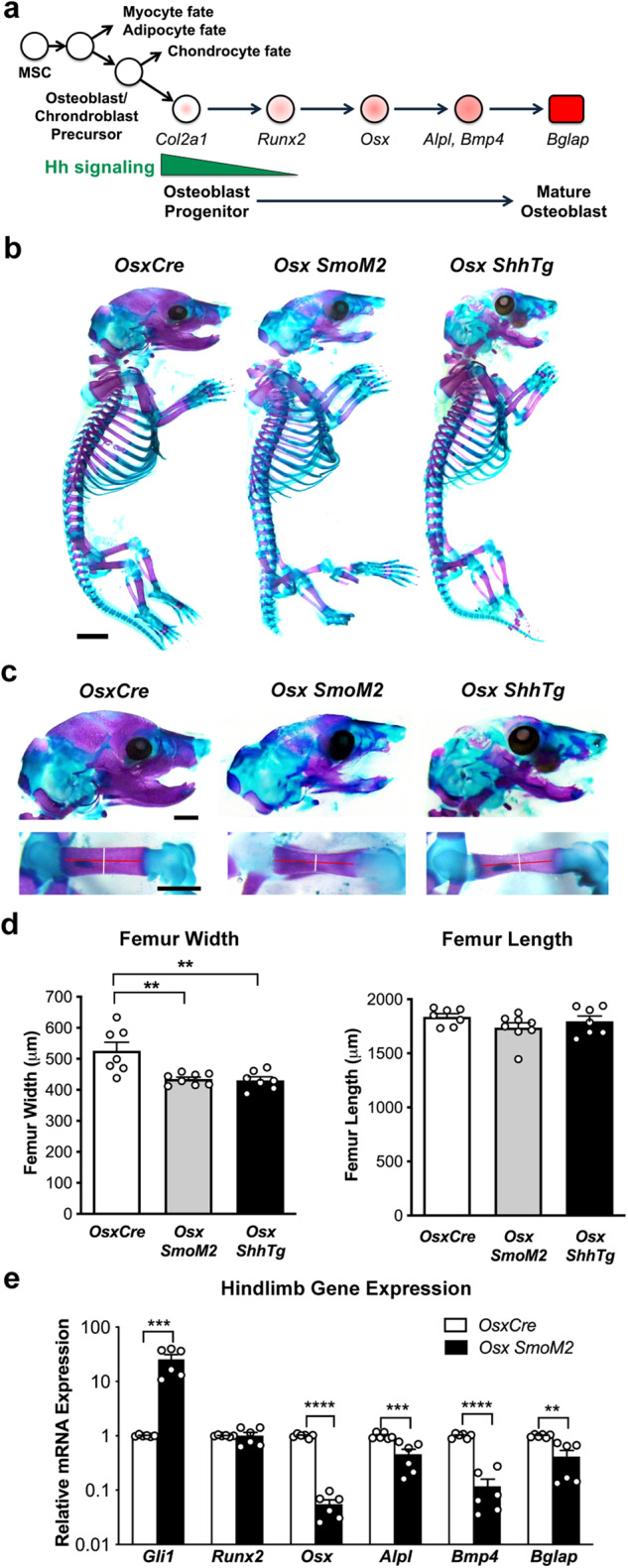
Activation of Hedgehog during mouse osteoblast development. **a** Schematic depicting osteoblast differentiation annotated with expression of relevant genes [27]. **b** Wholemount skeletal preparations stained with Alizarin Red (purple) and Alcian Blue (blue) of E18.5 *OsxCre*, *Osx SmoM2*, and *Osx ShhTg* embryos. Scale bar = 2 mm. **c** High magnification images of skull (top) and femur (bottom) from wholemount skeletal preparations of E18.5 *OsxCre*, *Osx SmoM2*, and *Osx ShhTg* embryos. Scale bar = 2 mm. Lines in femur images indicates measurement axis for width (white) and length (red). **d** Quantification of femur width (left) and length (right) from wholemount skeletal preparations. *n* = 7 independent embryos per genotype, mean ± SEM, **P < 0.01, one-way ANOVA/Tukey’s correction. **e** *Gli1, Runx2, Osx1, Alpl, Bmp4* and *Bglap* mRNA expression in *OsxCre*, and *OsxCre SmoM2* E18.5 hindlimbs. *n* = 6 independent embryos per genotype. All data are represented as mean + SEM, ** *P* < 0.01, *** *P* < 0.001, **** *P* < 0.0001, one-way ANOVA/Tukey’s correction.

Our findings in the mouse genetic osteosarcoma model that genetic inactivation of *Smo* prevents malignant tumorigenesis and drives an ossification phenotype led us to examine the function of the Hh signaling pathway in osteoblast differentiation in more detail. During normal osteoblast differentiation, IHH is secreted by the pre-hypertrophic chondroblasts, and is absolutely required for the specification of osteoblast progenitors from the osteoblast/chondroblast precursor cells (Fig. 6a) [27, 28]. However, following osteoblast progenitor specification Hh pathway activation is no longer required for the subsequent progression of osteoblast differentiation and is rapidly downregulated [10].

To investigate the consequences of sustained Hh pathway activation during osteoblast development we utilized the *OsxCre* mouse model, and two conditional gain of function Hh alleles, a constitutively active *Smo* allele [29] (*Osx SmoM2*), and a conditional transgenic allele that expresses SHH under the control of a CMV promoter [30] (*Osx ShhTg*). Analysis of skeletal development by wholemount alizarin red and alcian blue staining revealed widespread failure of intramembranous bone differentiation in E18.5 *Osx SmoM2* and *Osx ShhTg* embryos, characterized by reduced ossification of the calvarium, maxilla and mandible, and a significant reduction in femur width (Fig. 6b–d). Notably, endochondral ossification, required for the lengthening of long bones including the femur, was not affected (Fig. 6b–d). Early post-natal lethality of both models precluded further mature bone assessment. Analysis of Hh pathway activation in hind limbs of *Osx SmoM2* embryos revealed a 26-fold increase in *Gli1* mRNA (Fig. 6e) indicative of Hh pathway activation. Consistent with reduced ossification, markers of the osteoblast progenitor (*Osx*), preosteoblast (*Alpl, Bmp4*) and mature osteoblast (*Bglap*) were significantly decreased (Fig. 6e). These data suggest that sustained Hh pathway activation during osteoblast development maintains cells in an undifferentiated state leading to impaired ossification. Notably, genetic inactivation of *Trp53* (*Osx p53* KO), *Rb1* (*Osx Rb* KO) and both *Trp53* and *Rb1* (*Osx p53Rb* KO) in the osteoblast lineage also resulted in reduced ossification of the calvarium and mandible, and femur width compared to control littermates (Supplementary Fig. 10a, b), albeit to a lesser extent than that observed in the *Osx SmoM2* and *Osx ShhTg* embryos.

### Inhibition of Hh signaling inhibits osteosarcoma growth and drives differentiation

**Fig. 7:**
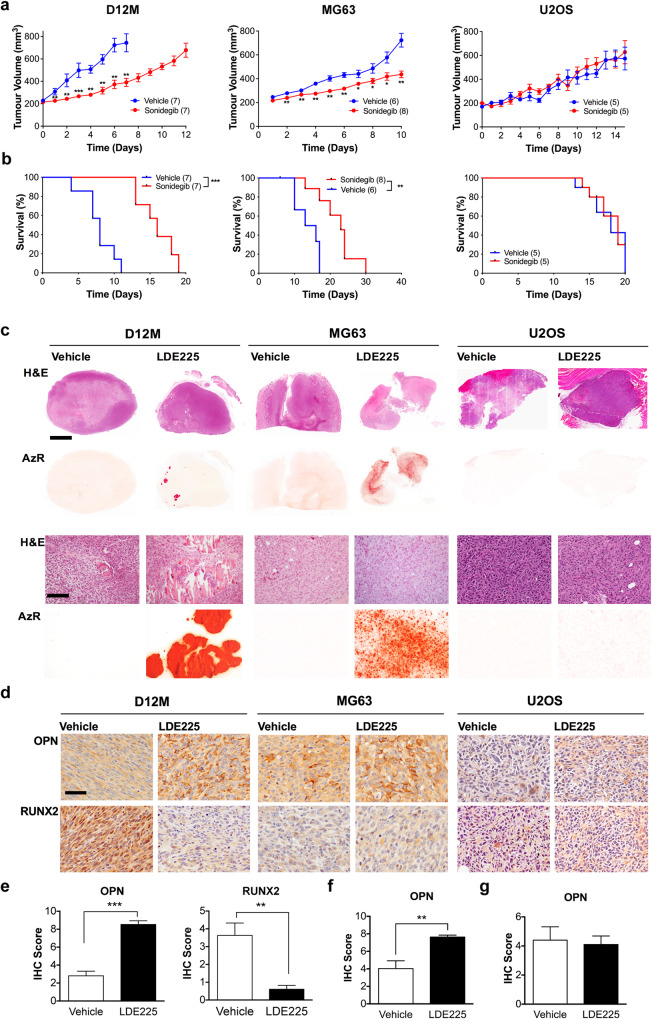
Effect of Smoothened inhibition on human and mouse osteosarcoma growth and differentiation in vivo. **a** Tumor volume of *p53Rb* KO mouse osteosarcoma allografts (D12M) and human osteosarcoma xenografts (MG63, U2OS) treated with vehicle control or 20 mg/kg sonidegib. All data are represented as mean ± SEM of biological replicates. *P < 0.05, **P < 0.01, ***P < 0.001, Mann–Whitney test. **b** Kaplan–Meier analysis of survival. **P < 0.01, ***P < 0.001, Log-rank (Mantel-Cox) test. **c** Hematoxylin and eosin, and alizarin red (AzR) staining of *p53Rb* KO mouse osteosarcoma allografts (D12M) and human osteosarcoma xenografts (MG63, U2OS) treated with vehicle control or 20 mg/kg sonidegib (LDE225). Scale bar = 2 mm (upper panels, scale bar = 200 µm (lower panels). **d** Osteopontin (OPN) and RUNX2 expression detected by immunohistochemistry in *Osx p53Rb* KO mouse osteosarcoma allografts (D12M) and human osteosarcoma xenografts (MG63, U2OS) treated with vehicle control or 20 mg/kg sonidegib (LDE225). Immunoperoxidase signal is shown in brown, counterstained with hematoxylin. Representative images are shown. Scale bar = 200 µm. **e** Quantification of OPN and RUNX2 immunohistochemistry in *Osx p53Rb* KO (D12M) allograft tumors. *n* = 4 independent animals. All data are represented as mean ± SEM of biological replicates. **P < 0.005; ***P < 0.001, Student’s unpaired *t* test. **f** Quantification of OPN immunohistochemistry in MG63 xenograft tumors. *n* = 4 independent animals. Data are represented as mean ± SEM, **P < 0.005, Student’s unpaired *t* test. **g** Quantification of OPN immunohistochemistry in U2OS xenograft tumors. *n* = 4 independent animals. Data are represented as mean ± SEM.

Based on our data from mouse bone development and osteosarcoma, we hypothesized that inhibition of SMO signaling in established osteosarcoma would result in tumor growth arrest and differentiation. To test whether this effect could be predicted based on *TP53* mutation status and primary ciliogenesis, we used in vivo flank osteosarcoma models generated from *Osx p53Rb* KO mice (D12M), ^45^Ca mice (CaOS18), and human MG63 and B143 (ciliated) and human U2OS and SJSA (unciliated) xenograft models. Treatment of tumor-bearing mice with the SMO inhibitor sonidgeib resulted in reduced tumor growth and significantly improved survival in both the D12M allograft and MG63 and B143 xenograft models, but not in mice with CaOS18, U2OS or SJSA tumors (Fig. 7a, b, Supplementary Fig. 11a, b). To further explore reverse paracrine signaling as the mechanism of Hh pathway activation in osteosarcoma tumor cells, as suggested by the in vitro data, we generated allograft models of D12M *Osx p53Rb* KO in which *Shh* was genetically inactivated (Supplementary Fig. 12). D12M *Shh* KO cells and tumors showed marked reduction in SHH expression and a similar response to sonidegib as lenti-control tumors suggesting that SHH secretion from stromal cells, not tumor cells, drives Hh pathway activation in osteosarcoma (Supplementary Fig. 12).

GLI2 expression, a marker of Hh pathway activation, was significantly reduced in D12M allografts and MG63 xenografts following sonidegib treatment but was scarcely detectable in both vehicle and sonidegib treated U2OS xenografts (Supplementary Fig. 13a–d). SHH expression and cilia frequency remained unchanged in sonidegib treated tumors in all models (Supplementary Figs. 13a, 14). Immunohistochemical analysis of PCNA revealed a significant reduction in proliferation in the D12M allograft and MG63 xenograft models, but not in the U2OS xenograft model (Supplementary Fig. 13a–d). In contrast, no change in cleaved Caspase-3 staining, a marker of apoptosis, was detected in any of the models (Supplementary Fig. 13a–d).

Histological analysis of tumor sections revealed focal areas of bone deposition in sonidegib treated tumors that was not apparent in vehicle controls and was confirmed by alizarin red staining (Fig. 7c). To further assess osteoblast differentiation, we performed immunohistochemical staining using the osteoblast progenitor maker, RUNX2, and the terminal osteoblast marker, osteopontin (OPN). In both D12M and MG63 tumors, sonidegib treatment resulted in an increase in OPN expression consistent with differentiation (Fig. 7d–g). Whilst RUNX2 expression was not detected in MG63 xenografts, vehicle treated D12M tumors exhibited strong RUNX2 expression that was significantly reduced in the sonidegib treated tumors (Fig. 7d, e). U2OS xenografts demonstrated no evidence of intratumoral mineralization or differential expression of RUNX2 and OPN (Fig. 7d, g). These results demonstrate that mutations in *TP53*, and primary cilia frequency is predictive of likely response to Hh pathway inhibition and support our conclusions that Hh signaling acts to prevent terminal differentiation in the setting of *TP53* mutation.

## Discussion

Genetic events leading to loss of either or both TP53 and RB1 function are common in human osteosarcoma [7, 31–36]. TP53 pathway inactivation through *TP53* structural variation, mutation, or amplification of *MDM2* is described in up to 97% of cases [7], while RB1 pathway disruption by *RB1* structural variation or mutation, or deletion of *CDKN2A*, occurs in >70% of cases [31, 35, 36]. More than 50% of osteosarcomas have alterations resulting in inactivation of both *TP53* and *RB1* [36]. A causal role for TP53 and RB1 in genetically modified mouse models of osteosarcoma [21], definitively implicates *TP53* and *RB1* in the pathogenesis of this disease.

The genetic and basic functional importance of ligand-dependent Hh signaling in cancer is not well understood. In genetically engineered cell culture and neural development models, we recently showed that combined inactivation of *Trp53* and *Rb1* disrupted transcriptional activation of autophagy, resulting in an increase in primary cilia formation [18]. This in turn led to a dramatic increase in canonical Hh signaling in response Hh ligand [18]. These results are in keeping with genetic requirements for *Smo* [37], *Shh* [38] and the ciliary kinesin *Kif3a* [18] in the initiation and progression of SCLC response to conditional deletion of *Trp53* and *Rb1* in the adult mouse airway epithelium.

Previous reports have suggested that Hh signaling may be a therapeutic target in osteosarcoma [12, 13, 15–17, 24]. The data presented here provides a clear mechanistic rationale with potential therapeutic implications by linking the loss of TP53 to activation of ligand-dependent Hh signaling in this disease. We show that loss of TP53 is associated with impaired autophagy, aberrant primary cilia formation and a dramatically enhanced response to Hh ligand stimulation. Our data confirm the work of others that expression of Hh ligands is commonly seen in human osteosarcoma [14, 15], which we further demonstrate in genetic and radiation induced mouse models. However, we now show the capacity of tumor cells to respond to Hh ligand is defined by genetic events that inactivate TP53. While previous studies, including our own, have implicated perturbation of RB1 signaling in Hh pathway activation in developmental and cancer models, we were unable to functionally show this by genetic inactivation experiments in the *Rb1* WT ^45^Ca osteosarcoma cell lines. However, re-expression of RB1 in an *Osx p53Rb* KO cell line did result in reduced cilia frequency and *Gli1* mRNA response that was comparable to re-repression of TP53, suggesting a role of RB1 in modulating Hh pathway response in this model. The reasons for this discrepancy between the ^45^Ca and *Osx p53Rb* KO osteosarcoma models are not clear but could be related to *Rb1* inactivation being a genetic driver of disease in the *Osx p53Rb* KO, but not in the ^45^Ca models. Notably, in addition to *RB1* loss of function events, RB1 signaling is also perturbed by commonly described point mutations and amplification or loss of upstream pathway regulators such as *CDKN2A, CDKN2B, CDKN2C*, *CDK4*, *CDK6*, *CCD2*. Loss of CDKN2A in ^45^Ca murine models and methylation of *CDKN2A* in a human osteosarcoma model was not associated with increased ciliogenesis or Hh response. It will be important to further define the precise impact of pathogenic *TP53* and *RB1* point mutations and altered expression of pathway regulators on Hh pathway activation using comprehensively genomically annotated models in future studies.

Autophagy is a dynamic process that has been implicated in the regulation of primary cilia assembly and function [18, 23, 39, 40]. Studies performed in mouse embryonic fibroblast cells (MEFs) have demonstrated opposing roles for autophagy in primary cilia assembly; (1) induction of cilia formation following acute serum starvation due to the degradation of the negative regulator of ciliogenesis, OFD1 [40]; and (2) inhibition of cilia formation and growth under basal and serum starved conditions by the degradation of the intraflagellar transport protein complex B protein, IFT20 [23]. Furthermore, in MEFs, the inactivation or knockdown of critical autophagy genes, including *Atg5*, leads to increased ciliogenesis and increased Hh pathway response [18, 23]. We have previously shown a link between TP53 and/or RB1, autophagy, ciliogenesis and Hh responsiveness in MEFs, murine developmental, and murine SCLC models all harboring genetic inactivation of *Trp53* and *Rb1* [18]. In this study we similarly show that in murine and human osteosarcoma cell lines with TP53 loss and abundance of primary cilia, there is an impairment of inducible autophagy, consistent with our previous results. However, loss of *Atg5* in a non-responsive, poorly ciliated ^45^Ca osteosarcoma cell line does not result in changes to cilia frequency or *Gli1* mRNA. The reason for the disconnect between autophagy and ciliogenesis in this ^45^Ca cell line is unknown. Since previous studies describing this mechanism have been performed in mostly genetically homogeneous models it is possible that the undescribed genomic heterogeneity of the ^45^Ca tumor models may have unexpected impacts on cell homeostasis. The complex and dynamic nature of autophagy is known to depend on spatial, temporal and cell-specific contexts. Further, systematic interrogation of the effects of autophagy and ciliogenesis in defined osteosarcoma models will be required to understand interactions between autophagy and cilia assembly and maintenance in different genetic contexts, including on the background of TP53 and RB1 loss.

The developmental context of our results provides important insight into the potential importance of Hh ligand signaling in osteosarcoma. Genetic deletion of *Ihh* resulted in a complete failure of endochondral bone formation [11], whereas conditional deletion of *Smo* in maturing osteoblasts resulted in normal bone development [10]. These results suggest that Hh signaling through SMO is involved in the early specification of primitive osteoblast cells [10]. For the first time, we now show that persistent activation of Hh signaling in osteoblast precursors defined by expression of *Osx* impairs intramembranous ossification and resulted in a gene expression profile consistent with a primitive osteoblast phenotype. These data strongly suggest that persistent Hh signaling effectively prevented the developmental transition from a primitive to mature osteoblast phenotype.

To date, the strongest evidence supports these Hh-dependent primitive osteoblast cells as the cell of origin in osteosarcoma [21, 41, 42]. Consistent with this idea, we further demonstrate that deletion of *Smo* along with both *Trp53* and *Rb1* in *Osx* expressing cells blocks osteosarcoma formation, and instead resulted in the formation of highly differentiated benign bone tumors. This finding suggests that Hh signaling is epistatic to loss of two potent tumor genes, and that continued Hh signaling is required to produce an undifferentiated, malignant phenotype. Furthermore, this result provides a firm genetic basis for testing SMO antagonists as potential therapeutics in osteosarcoma. Notably, in this study we focused on canonical Hedgehog pathway signaling via HH-SMO-GLI. However, loss of ciliary IFT80 in osteoblast precursor cells has been reported to inhibit ciliogenesis, block canonical Hh signaling by disrupting SMO ciliary localization, and elevate non-canonical HH-SMO-Gαi-RhoA signaling [43]. The switch from canonical to non-canonical Hh signaling, determined by a failure to elevate *Gli1* and consequently promote RhoA activity, was observed when cilia frequency was reduced to 30% in osteoblast precursor cells [43]. Whether the subcellular localization of SMO similarly effects the balance of canonical versus non-canonical Hh signaling in osteosarcoma is unknown. The lack of significant transcriptional upregulation of common canonical Hh pathway target genes, including *GLI1*, in the moderately ciliated human osteosarcoma cell lines, all with a cilia frequency <20%, suggests that similar non-canonical Hh signaling mechanisms will be important to investigate.

Human osteosarcomas are often characterized by an immature phenotype and the expression of primitive osteoblast markers, indicative of a developmental block. Our demonstration of in vivo growth arrest and the expression of markers of terminal osteoblast differentiation in TP53 mutant osteosarcoma models in response to the SMO antagonist sonidegib support this notion. Sonidegib is FDA approved for locally advanced basal cell carcinoma and is currently in clinical trials for numerous other hematological and solid tissue malignancies [44]. In a Phase I dose escalation study in children with advanced solid tumors potentially dependent on Hh signaling, sonidegib was shown to be well tolerated and antitumor activity was restricted to patients exhibiting a Hh pathway activated signature [45]. Notably, a small cohort of five osteosarcoma patients were included in the study but none demonstrated a Hh pathway activated signature or response to treatment. More recently, a Phase II study evaluating the efficacy and safety of sonidegib in adult patients with advanced/metastatic sarcomas, including an osteosarcoma cohort has recently concluded and the results are eagerly anticipated (ACTRN12612000533897). Taken together, our results have two important translational implications. First, human osteosarcomas that are likely to respond to SMO antagonists will be limited to tumors with genetic *TP53* inactivation, and that express SHH or IHH and primary cilia. Second, SMO antagonists have potential as differentiative agents rather than as cytotoxic therapy.

## Materials and methods

### Animal experiments

B6;129-Gt(ROSA)26Sor^tm1(cre/ERT)Nat/J^ (*ESRCre* [46]), B6.129P2-Trp53^tm1Brn/J^ (*Trp53*^*lox/lox*^ [47]), B6;129-Rb1^tm3Tyj/J^ (*Rb1*^*lox/lox*^ [48]), Smo^tm2Amc/J^ (*Smo*^*lox/lox*^ [28]) and B6.Cg-Tg(Sp7-tTA,tet0- EGFP/cre)1Amc/J (*OsxCre* [10]), *Gt(ROSA)26Sor*^*tm1(Smo/EYFP)Amc*/J^ (*SmoM2* [29]), and C57BL/6J – (ROSA26)ShhTgW (*ShhTgW* [30]) alleles have been described previously. All mice were backcrossed onto and maintained on a C57BL/6J background. Inbred C57BL/6J were obtained from the Monash Animal Research Platform and NOD.Cg-*Prkdc*^*scid*^*Il2rg*^*tm1Wjl*^/SzJ (NSG) and BALB/c- Fox1nu/Ausb (BALB/c Nude) mice were purchased from Australian BioResources.

### Genetic mouse model of osteosarcoma

Male and female *OsxCre*, *OsxCre;Trp53*^*lox/lox*^*;Rb1*^*lox/lox*^, *OsxCre;Trp53*^*lox/lox*^*;Rb1*^*lox/lox*^*;Smo*^*lox/lox*^ mice were monitored for signs of palpable and progressive tumor development, body weight loss >10% and signs of distress. At the completion of the study, mice were euthanized in a carbon dioxide chamber, imaged using the Feinfocus Y. Cougar Microfocus X-ray Inspection System (YXLON) and tissues harvested for cell line generation, pathological examination and immunohistochemistry. Confirmation of allele recombination in tumor tissue was performed using genomic PCR according to the standard JAX protocols and using oligonucleotide sequences described in Supplementary Table 1.

### Osteosarcoma allografts and xenografts

A total of 1 × 10^6^ *Osx p53Rb* KO (D12M), MG63, U2OS, B143, SJSA, CaOS18, D12M lenti-control and D12M SHH KO cells were injected into the flanks of 6–8 week old female NSG or BALB/c Nude mice. Cells were resuspended in 100 μl of 1:1 mixed cell suspension in PBS and Matrigel. Tumor size was measured daily using digital calipers, and volumes calculated according to the formula: Tumor Volume (mm^3^) = (Width^2^ × Length)/2. Once tumors reached a volume of 200 mm^3^ mice were randomized to receive the SMO inhibitor sonidegib (LDE225) (20 mg/kg) or vehicle control (0.5% Methylcellulose/0.5% Tween 80). Mice were treated daily by oral gavage until ethical endpoints were reached. Ethical endpoints were defined as a tumor volume of 800 mm^3^ or greater, a body weight loss of >10% or signs of general distress. At the completion of the study, mice were euthanized in a carbon dioxide chamber and tissue was harvested from the flanks for histology and analysis of differentiation markers.

All mice used in this study were housed under SPF conditions with a standard day/night cycle and fed *ad libitum*. All experiments involving animals were approved in advance by the Animal Ethics Committee at Monash University (Ethics Number: MMCA2012/24, MMCA2015/11, MMCA2015/12, MMCA2015/13, MMCA2015/41, MMCA2019/19) and were carried out in accordance with the Australian Code of Practise for the Care and Use of Animals for Scientific Purposes. All animal experiments were unblinded.

### Human osteosarcoma cell lines

Authenticated U2OS, SJSA, B143, HOS and MG63 human osteosarcoma cell lines were obtained from ATCC and maintained in DMEM (Gibco, Invitrogen) supplemented 10% FCS, 100 U/mL penicillin, and 10 mg/mL streptomycin in a humidified 5% CO2 /95% air atmosphere at 37 °C. All cell lines were re-authenticated in our laboratory immediately prior to experimentation and routinely tested for mycoplasma (all negative). Cells were cultured to approximately 70% confluence and for no longer than 20 passages in total for any experiment.

### Mouse osteosarcoma cell lines

Radiation-induced and conditional genetic mouse osteosarcoma cell lines were maintained in alpha-MEM supplemented with 10% FCS and 100 U/mL penicillin, and 10 mg/mL streptomycin in a humidified 5% CO2 /95% air atmosphere at 37 °C.

For development of isogenic mouse osteosarcoma cell lines, Two independent guide RNAs (Supplementary Table 2) were cloned into Lenti CRIPSRv2 plasmid containing Cas-9 and puromycin selection marker in the backbone. Guide RNA oligos were cloned into the plasmid and validated using the previously described protocol [49]. Plasmids were transfected into HEK293T cells to generate viral particles, which were transduced into selected mouse osteosarcoma cell lines. Puromycin (5 μg/ml) was added after 48 h for selection of transduced cells. Single cells were sorted into a 96-well plate and were expanded and validated using western blot. Knockdown experiments using siRNA (Supplementary Table 2) were performed as described in [18].

### Ligand and inhibitor treatment

To evaluate response to Hh ligand using *Gli1* mRNA expression, mOS cells were seeded at 100,000 cells/well on 6 well plates and grown to 80–90% confluence in alpha-MEM with 10% FCS. Cells were treated in media containing 0.2% FCS, with PBS only, 1 μg/mL rhSHH and/or 400 nM of sonidegib, a dose previously shown to abolish ligand-dependent Hh signaling in MEFs and SCLC cell lines [18]. After 24 h of treatment, RNA was extracted using Qiagen RNA Mini-Kit. For autophagy analysis, mOS and hOS cells were seeded in 10 cm culture dishes at a density of 1 × 10^6^. At about 80% confluence, cells were treated with 50 μM of chloroquine or 50 nM Bafilomycin A with either 10% FCS media or serum free media for 24 h. Post treatment, whole cell lysates were extracted in RIPA buffer.

Expression of TP53 and RB1 in the *Osx p53Rb* KO mouse osteosarcoma cell line, D12M, was performed and assessed as described in [18].

### RNA extraction and gene expression

RNA was prepared using the Qiagen RNeasy Mini Kit and cDNA generated using the first-strand Superscript III synthesis kit. Real-time qRT-PCR was performed in 384-well plate using SYBR Green master mix on a Quantstudio 6 Real Time PCR System (Applied Biosystems) using custom designed primers for mouse *B2m*, *Ptch1, Smo, Gli1, Gli2, Hhip, Ccnd1, Runx2*, *Osx, Alpl, Col1a1, Ebf2, Bmp4, Ibsp, Bglap, Ihh, Dhh, Shh, Cdk1, Ccne1, Cdc6* and human *IHH, DHH, SHH, GLI1, PTCH1, HHIP, CDK1, CCNE1, CDC6* (oligonucleotide sequences: Supplementary Table 3). Primers were diluted to a final concentration of 250 nM with 6 µL of SYBR Green mastermix and 4 µL of cDNA in a 384-well plate. Samples were run in triplicates and transcript levels relative to β2 M were calculated using a standard curve method.

For RNA-seq of U2OS and MG63, RNA was isolated from cells treated with 1 µg/ml rhSHH or PBS for 24 h. The samples were subjected to DNBSEQ eucaryotic strand-specific mRNA library preparation and paired-end sequenced using the DNBseq platform to a depth of 23 million reads per sample (BGI Genomics). The reads were aligned to reference genome hg38 and counts performed using Galaxy Australia. Differentially expressed genes were analysed using Degust platform at Monash University. RNA-seq data are available through GEO, GSE192547.

### Western blotting

Western blots were performed according to the methods described in [18] (antibody details: Supplementary Table 4). For analysis of autophagy flux, densitometric analysis of the LC3-II and Actin bands were performed using Odyssey Infrared Imaging System software as previously described [18, 23]. To determine the autophagic flux ratio, the serum-starved autophagic flux value was divided by the 10% serum autophagic flux value.

### Detection of primary cilia

Primary cilia staining was performed as previously described [18] (antibody details: Supplementary Table 5). For localization of SMO to the primary cilia, human osteosarcoma cells were plated on 14 mm circular coverslips in a 24 well plate in DMEM supplemented with 10% FCS. Once cells reached 80% confluency media was changed to DMEM with 0.2% FCS. Following 24 h culture under serum starved conditions, cells were treated with 1 µg/ml rhSHH and/or 400 nM sonidegib for a further 24 h before rinsing in PBS and fixing in 10% buffered formalin. Fixed wells were washed three times in 1xPBS then permeabilized in 0.1% Triton X-100 (Sigma-Aldrich, X100–500 ML)/1xPBS for 20 min. Cells were then incubated in 1% SDS for 5 min, before re- fixing in 10% buffered formalin for a further 10 min. After three washes in 1xPBS cells were blocked in in Odyssey blocking buffer (LI-COR Biosciences: 927–4000) for 15 min. Rabbit polyclonal anti-SMO (1:50, LifeSpan BioSciences, LS-A2668) and mouse monoclonal anti-acetylated tubulin (1:200, Sigma-Aldrich, T7451) pre-labeled using a Mix-n-stain antibody labeling kit (Biotium, #9952) were incubated with cells for 2 h at room temperature, before washing in 1xPBS and mounting in ProLong™ Gold Antifade Mount with DAPI (ThermoFisher Scientific: P36931) on Superfrost slides.

Coverslips were viewed using an Eclipse Ti-E Nikon C1 Inverted Research Confocal Microscope equipped with a 60x oil immersion objective with identical gain, offset and laser power settings. High-resolution images were acquired using the NIS Elements Confocal Imaging (Nikon) software. The percentage of ciliated cells and total was counted in five fields of vision per cell line and/or treatment group to determine primary cilia frequency.

### Genomic sequencing

Sanger sequencing of *Trp53* and *Rb1* in mouse osteosarcoma cell lines was performed on cDNA PCR product (oligonucleotide sequences: Supplementary Table 6). PCR products were purified using Wizard PCR and gel Clean-up kit (Promega) and submitted to the MHTP Medical Genomics Facility for sequencing using forward and reverse primers. Sequence were aligned to reference using SeqMan Pro (DNASTAR, Lasergene, Version 8.1.5).

Detection of *Cdnk2a* deletion in mouse osteosarcoma cell lines was performed on cDNA PCR product (oligonucleotide sequences: Supplementary Table 6). PCR products were separated by gel electrophoresis on a 1.5% agarose gel stained with SYBR® Safe DNA gel stain. A 1 kb Plus DNA ladder was used to interpret band sizes. Gels were visualized under a Doc-ItTM 210 imaging system (UVP). Exposure was adjusted for optimal visualization.

### Generation of embryos for analysis of skeletal development

*OsxCre* males were time-mated with *SmoM2* and *ShhTgW* females, *OsxCre;Trp53*^*lox/lox*^ males were time mated with *Trp53*^*lox/lox*^ females, *OsxCre;Rb1*^*lox/lox*^ males were time mated with *Rb1*^*lox/lox*^ females, and *OsxCre;Trp53*^*lox/lox*^*;Rb1*^*lox/lox*^ males were time mated with *Trp53*^*lox/lox*^*;Rb1*^*lox/lox*^ females, and checked for vaginal plugs each morning. At E18.5, pregnant females were humanely sacrificed, uterine horns were removed, and embryos isolated for genotyping, skeletal preparations and RNA extractions.

### Skeletal preparations

E18.5 embryos were dissected, eviscerated and skin was removed. Embryos were then placed in a 15 mL falcon tube and fixed in 100% ethanol for 24 h at room temperature on a rocker. Embryos were transferred to 100% acetone for 24 h at room temperature on a rocker. Embryos were stained with Alcian Blue/Alizarin Red stain for 3–4 days at 37 °C on a rocker. Embryos were rinsed in ddH_2_O. Embryos were then transferred to a 1% KOH solution and incubated at RT on a rocker for a minimum of 3 h. Embryos were transferred into fresh 1% KOH and incubated overnight at room temperature on a rocker. Embryos were then placed through the following series of glycerol/KOH washes:

20% glycerol/1% KOH (24 h at room temperature on a rocker) 50% glycerol/1% KOH (24 h at room temperature on a rocker) 80% glycerol/1% KOH (24 h at room temperature on a rocker). Embryos were then stored at room temperature in 80% glycerol/1% KOH and photographs were then taken. Femur length and width analysis was measured using FIJI software.

### Immunohistochemistry

Immunohistochemical analysis of SHH in osteosarcoma patient tissue, radiation induced and genetic mouse osteosarcoma and xenografts, and SHH, GLI2, PCNA, cleave Caspase-3, OPN and RUNX2 staining on osteosarcoma allograft and xenografts was performed on paraffin embedded sections as previously described [38] (antibody details: Supplementary Table 7). Quantification of staining was performed using the multiplicative quickscore method by a blinded observer [50]. Pathological analysis of H&E sections was blindly performed by a pathologist.

### Alizarin red staining

To visualize calcium deposits in osteosarcoma allograft and xenograft tumor tissue, 2 g of alizarin red powder was dissolved in 100 mL of distilled water and adjusted to a pH of 4.1–4.3 with 0.5% of ammonium hydroxide. Slides were dewaxed in Histosol, hydrated into 70% ethanol and briefly rinsed in distilled water. Alizarin red solution was applied for 30 s to sections and excess dye was blotted off. Slides were then dipped in acetone 20 times, dipped in a 1:1 Acetone-Histosol solution 20 times, then finally cleared in Histosol for 5 min. Slides were then mounted onto coverslips using DPX.

### Quantification and statistical analysis

All data were analyzed with GraphPad Prism (version 9.5.1) and represented as mean + SEM. A paired-two tail *t* test was used for two samples with a single variable. A one-way ANOVA followed by a Tukey’s multiple comparison test was used for more than two samples with one variable. Log- rank (Mantel-Cox) test was used for comparison of Kaplan–Meier survival curves. A *p* value of less than 0.05 was considered statistically significant and is denoted by * < 0.05, ** < 0.01, *** < 0.001, **** < 0.0001. The number of samples (‘n’) used for calculating statistics is indicated in the Figures or accompanying legends.

## Supplementary information

Supplementary Information

Supplementary Figs. 1–14

Supplementary Table 1

Supplementary Table 2

Supplementary Table 3

Supplementary Table 4

Supplementary Table 5

Supplementary Table 6

Supplementary Table 7

## Data Availability

All relevant data is available from the authors upon request. RNA-seq data are available through GEO, GSE192547.
