# Supplementary material for: Ligand-dependent hedgehog signaling maintains an undifferentiated, malignant osteosarcoma phenotype
Source: Oncogene. 2023 Oct 16;42(47):3529–41. doi: 10.1038/s41388-023-02864-7 (PMC10656285; doi:10.1038/s41388-023-02864-7)
Supplement: Supplementary file 1 — Supplementary Information [file 41388_2023_2864_MOESM1_ESM.docx]

**Supplementary Fig. 1 Hedgehog ligand expression in mouse osteosarcoma cells. a** Quantification of SHH expression in mouse osteosarcoma cell lines as shown in Fig. 1a. *n* = 5 independent experiments, mean + SEM. **b** *Ihh, Dhh* and *Shh* mRNA expression in individual ^45^Ca and *p53Rb* KO mouse osteosarcoma cell lines. Values are normalized to the expression of *β2-microglobulin.* *n* = 3, mean + SEM, **P<0.05, **P<0.01, ****P<0.0001,* one-way ANOVA/Tukey’s test. **c** Average *Ihh, Dhh* and *Shh* mRNA expression in ^45^Ca and *p53Rb* KO mouse osteosarcoma cell lines. Values are normalized to the expression of *β2-microglobulin.* *n* = 5 independent cell lines, mean + SEM, ***P<0.01,* one-way ANOVA/Tukey’s test. **d** *Cdk1, Ccne1* and *Cdc6* mRNA expression in Cdkn2a null (CaOS18) and WT (CaOS18) ^45^Ca and mouse osteosarcoma cell lines. Values are normalized to the expression of *β2-microglobulin.* *n* = 3, mean + SEM.

**Supplementary Fig. 2 Effects of Cdkn2a and Rb1 inactivation in ^45^Ca mouse osteosarcoma cells. a** Western blot analysis of CDKN2A and ACTIN expression in the radiation induced (^45^Ca), CaOS30 mouse osteosarcoma cell lines following lenti-viral transduction of *Cdkna* gRNAs and single cell cloning. **b** *Gli1* mRNA expression in CaOS30 isogenic cell lines treated with PBS vehicle control, 1μg/ml rhSHH, 1μg/ml rhSHH and 400nM sonidegib (LDE225) or 400nM sonidegib for 24 hours. Values are normalized to the expression of *β2-microglobulin.* *n* = 3, one-way ANOVA/Tukey’s test. **c** Representative images demonstrating immunofluorescence colocalization of acetylated α-tubulin (AcTUB) and ARL13B in CaOS30 isogenic cell lines cultured in 10% serum, or serum-free media for 24 hours. Scale bar, 5µm. **d** Primary cilia frequency in CaOS30 isogenic cell lines cultured in 10% serum or serum-free media for 24 hours. *n* = 5 individual replicates per cell line, mean + SEM, Student’s unpaired *t*-test. **e** Western blot analysis of RB1 and ACTIN expression in the ^45^Ca, CaOS28 and CaOS30 mouse osteosarcoma cell lines following lenti-viral transduction of *Rb1* gRNAs and single cell cloning. **f** *Gli1* mRNA expression in CaOS28 isogenic cell lines treated with PBS vehicle control, 1μg/ml rhSHH, 1μg/ml rhSHH and 400nM sonidegib (LDE225) or 400nM sonidegib for 24 hours. Values are normalized to the expression of *β2-microglobulin.* *n* = 3, one-way ANOVA/Tukey’s test. **g** *Gli1* mRNA expression in CaOS30 isogenic cell lines treated with PBS vehicle control, 1μg/ml rhSHH, 1μg/ml rhSHH and 400nM sonidegib (LDE225) or 400nM sonidegib for 24 hours. Values are normalized to the expression of *β2-microglobulin.* *n* = 3, one-way ANOVA/Tukey’s test. **h** Representative images demonstrating immunofluorescence colocalization of acetylated α-tubulin (AcTUB) and ARL13B in CaOS28 isogenic cell lines cultured in 10% serum, or serum-free media for 24 hours. Scale bar, 5µm. Quantification of primary cilia frequency in CaOS28 isogenic cell lines cultured in 10% serum or serum-free media for 24 hours. *n* = 5 individual replicates per cell line, mean + SEM, ***P<0.01, ****P<0.0001,* Student’s unpaired *t*-test. **i** Representative images demonstrating immunofluorescence colocalization of acetylated α-tubulin (AcTUB) and ARL13B in CaOS30 isogenic cell lines cultured in 10% serum, or serum-free media for 24 hours. Scale bar, 5µm. Quantification of primary cilia frequency in CaOS30 isogenic cell lines cultured in 10% serum or serum-free media for 24 hours. *n* = 5 individual replicates per cell line, mean + SEM*,* Student’s unpaired *t*-test.

**Supplementary Fig. 3 Restoration of p53 and Rb function in *Osx p53Rb* KO mouse osteosarcoma cells.** **a** FACS analysis of *Osx p53Rb* KO mouse osteosarcoma cell line, D12M, transduced with an empty vector, or vectors expressing p53-GFP, Rb-mCherry or both p53-GFP and Rb-mCherry fusion proteins. **b** Western blot analysis of TP53, RB1 and ACTIN expression in *Osx p53Rb* KO mouse osteosarcoma cells transduced with an empty vector, or vectors expressing p53-GFP, Rb-mCherry or both p53-GFP and Rb-mCherry constructs. **c** *Gli1* expression in *Osx p53Rb* KO mouse osteosarcoma cells transduced with an empty vector control, or vectors expressing p53-GFP, Rb-mCherry or both p53-GFP and Rb-mCherry fusion proteins, then treated with PBS or 1ug/ml rhSHH for 24 hours. Values are normalized to the expression of *β2-microglobulin.* *n* = 3 independent replicates, mean + SEM, ****P<0.001, ****P<0.001,* one-way ANOVA/Tukey’s test. **d** Primary cilia frequency in untransduced *Osx p53Rb* KO mouse osteosarcoma cells cultured in serum-free conditions for 24 hours, and following transduction with vectors expressing p53-GFP, Rb-mCherry or both p53-GFP and Rb-mCherry fusion proteins. *n* = 4 individual replicates, mean + SEM, *****P<0.001*, one-way ANOVA/Tukey’s test. **e** Confocal immunofluorescence detection of GFP, mCherry and ARL13B in a *Osx p53Rb* KO mouse osteosarcoma cell line either untransduced of transduced with lentiviral vectors expressing p53-GFP, Rb-mCherry or both p53-GFP and Rb-mCherry fusion proteins. Scale bar = 5um.

**Supplementary Fig. 4 Autophagic flux in ^45^Ca and *p53Rb* KO mouse osteosarcoma cell lines. a** Western blot analysis of LC3 and ACTIN expression in Hh non-responsive, *Trp53* mutant and *Rb1* WT CaOS18, and Hh responsive, *Trp53* null and *Rb1* WT CaOS25 and *Trp53* null and *Rb1* null C78F cell lines cultured in 10% serum or serum free media with or without 50µM chloroquine (CHQ) for 24 hours. *n* = 4 independent experiments. **b** Quantification of autophagic flux by western blot in mouse osteosarcoma cell lines as shown in **a**. *n* = 4 independent experiments, mean + SEM. Graph represents fold change in autophagic flux in serum-free media (induced autophagy) compared to normal serum (basal autophagy) ***P* < 0.01, ****P* < 0.001, one-way ANOVA/Bonferroni correction.

**Supplementary Fig. 5 Effects of *Atg5* loss in ^45^Ca mouse osteosarcoma cells. a** Western blot analysis of CDKN2A and ACTIN expression in the radiation induced (^45^Ca), CaOS18 mouse osteosarcoma cell lines following lenti-viral transduction of lenti-control or *Atg5* gRNAs. *Gli1* mRNA expression in CaOS18 isogenic cell lines treated with PBS vehicle control or 1μg/ml rhSHH for 24 hours. Values are normalized to the expression of *β2-microglobulin.* *n* = 3, mean + SEM, **P<0.05*, two-way ANOVA/Tukey’s test. Representative images demonstrating immunofluorescence colocalization of acetylated α-tubulin (AcTUB) and ARL13B in CaOS18 isogenic cell lines cultured in 10% serum, or serum-free media for 24 hours. Scale bar, 5µm. Quantification of primary cilia frequency in CaOS18 isogenic cell lines cultured in 10% serum or serum-free media for 24 hours. *n* = 5 individual replicates per cell line, mean + SEM, Student’s unpaired *t*-test. **b** Western blot analysis of CDKN2A and ACTIN expression in the radiation induced (^45^Ca), CaOS18 mouse osteosarcoma cell lines following transfection with siNTC or si*Atg5*. *Gli1* mRNA expression in CaOS18 cell lines following transfection with siNTC and si*Atg5* treated with PBS vehicle control or 1μg/ml rhSHH for 24 hours. Values are normalized to the expression of *β2-microglobulin.* *n* = 3, mean + SEM, two-way ANOVA/Tukey’s test. Representative images demonstrating immunofluorescence colocalization of acetylated α-tubulin (AcTUB) and ARL13B in CaOS18 cells transfected with siNTC or si*Atg5* cultured in 10% serum, or serum-free media for 24 hours. Scale bar, 5µm. Quantification of primary cilia frequency in CaOS18 isogenic cell lines cultured in 10% serum or serum-free media for 24 hours. *n* = 5 individual replicates per cell line, mean + SEM, ***P<0.01, ****P<0.0001*, two-way ANOVA/Tukey’s test.

**Supplementary Fig. 6 Genetic recombination of *Trp53, Rb1* and *Smo* alleles conditional genetic knockout mouse model of osteosarcoma.** Genomic PCR for *OsxCre* (**a**), *Trp53* (**b**), *Rb1* (**c**) and *Smo* (**d**) alleles in *Osx p53Rb* KO and *Osx p53RbSmo* KO osteosarcoma and osteoid osteoma tissue.

**Supplementary Fig. 7 Hedgehog signaling, osteoblast differentiation and primary cilia in response to targeting SMO in the *Osx p53Rb* KO model. a** *Smo, Ptch1, Gli1, Gli2, Hhip* and *Ccnd1* mRNA expression in *Osx p53Rb* KO (*n = 6* independent animals) and *Osx p53RbSmo* KO (*n = 5* independent animals) tumor tissues. All data are represented as mean + SEM, * *P* < 0.05, Student’s unpaired t-test. **b** Expression of *Runx2, Alpl, Col1a1, Ebf2, Bmp4, Ibsp* and *Bglap* mRNA in *Osx p53Rb* KO (*n = 5* independent animals) and *Osx p53RbSmo* KO (*n = 5* independent animals) tumor tissues. All data are represented as mean + SEM. **c** Alizarin red staining of *p53Rb* KO mouse osteosarcoma cell line (C78F) treated with vehicle control or 400nM sonidegib (LDE225) for 21 days *in vitro*. Scale bar = 2.5mm. **d** *Gli1, Runx2, Alp, Col1a1, Ebf2, Bmp4, Ibsp* and *Bglap* mRNA expression in *p53Rb* KO mouse osteosarcoma cell line (889M) treated with vehicle control or 400nM sonidegib (LDE225) for 21 days *in vitro.* *n = 3* independent replicates. All data are represented as mean + SEM, * *P* < 0.05, Student’s unpaired t-test. **e** SHH expression detected by immunohistochemistry in tumor sections from *Osx p53RbSmo* KO mouse osteomas. Immunoperoxidase signal is shown in brown, counterstained with hematoxylin. Scale bar = 40µm. **f** Immunofluorescence colocalization of ARL13B in *Osx p53RbSmo* KO mouse osteoma. Scale bar = 15µm. **g** Primary cilia abundance in *Osx p53RbSmo* KO mouse osteoma. *n* = 5 independent osteomas, mean + SEM.

**Supplementary Fig. 8 Hedgehog signaling in human and mouse osteosarcoma *in vivo*. a** SHH expression detected by immunohistochemistry in human osteosarcoma samples using two independent antibodies (SC9024, N term; and Ab53281 C term). Immunoperoxidase signal is shown in brown, counterstained with hematoxylin. HH (-) depicts IgG control-stained section, HH (+) is stained with Ab53281. Representative images are shown. Scale bar = 200µm. **b** Quantitation of SHH immunohistochemistry, correlating the IHC scores using the two independent SHH antibodies. **c** SHH expression detected by immunohistochemistry in *p53Rb* KO (D12M) allografts. Immunoperoxidase signal is shown in brown, counterstained with hematoxylin. Representative image is shown. Scale bar = 50µm.

**Supplementary Fig. 9 Hedghog signaling and autophagic flux in human osteosarcoma cell lines *in vitro*. a** *IHH, DHH* and *SHH* mRNA expression in individual human osteosarcoma cell lines. Values are normalized to the expression of *β2-microglobulin.* *n* = 3, mean + SEM, **P<0.05, **P<0.01,* one-way ANOVA/Tukey’s test. **b** Average *IHH, DHH* and *SHH* mRNA expression in human osteosarcoma cell lines. Values are normalized to the expression of *β2-microglobulin.* *n* = 5 independent cell lines, mean + SEM. **c** *CDK1, CCNE1* and *CDC6* mRNA expression in human osteosarcoma cell lines. Values are normalized to the expression of *β2-microglobulin.* *n* = 3, mean + SEM. **P<0.05, **P<0.01, ***P<0.001,* one-way ANOVA/Tukey’s test. **d,e** Expression of *GLI1, HHIP, BCL2, FOXL1* and *PRDM1* in U2OS (**d**) and MG63 (**e**) cells treated with PBS or 1ug/ml rhSHH for 24 hours. n = 3 individual replicates, mean + SEM. **f** *GLI1, PTCH1* and *HHIP* mRNA expression in U2OS, SJSA, B143, HOS and MG63 human osteosarcoma cell lines treated with PBS vehicle control, 1μg/ml rhSHH, 1μg/ml rhSHH and 400nM sonidegib or 400nM sonidegib for 24 hours. Values are normalized to the expression of *β2-microglobulin.* *n* = 3, mean + SEM, ***P<0.01, ***P<0.001,* one-way ANOVA/Tukey’s test. **g** Western blot analysis of LC3 and ACTIN expression in non-ciliated (U2OS) and ciliated (MG63) human osteosarcoma cell lines cultured in 10% serum or serum-free with or without 50µM chloroquine (CHQ) for 24 hours. *n* = 4 independent experiments. **h** Quantification of autophagic flux by western blot in mouse osteosarcoma cell lines as shown in **a**. *n* = 4 independent experiments, mean + SEM. Graph represents fold change in autophagic flux in serum-free media (induced autophagy) compared to normal serum (basal autophagy) **P* < 0.05, Student’s unpaired *t*-test.

**Supplementary Fig. 10 Activation of Hedgehog during mouse osteoblast development. a** High magnification images of skull (top) and femur (bottom) from E18.5 wholemount skeletal preparations stained with Alizarin Red (purple) and Alcian Blue (blue) of E18.5 Control, *Osx p53* KO, *Osx Rb* KO and *Osx p53Rb* KO embryos. Scale bar = 1mm. Lines in femur images indicates measurement axis for width (white) and length (red). **b** Quantification of femur width (left) and length (right) from wholemount skeletal preparations. *n* = 6 independent embryos per genotype, mean + SEM, ***P* < 0.01, *****P* < 0.0001, one-way ANOVA/Tukey’s correction.

**Supplementary Fig. 11 Effect of Smoothened inhibition on human and mouse osteosarcoma growth *in vivo.*** **a** Tumor volume of ^45^Ca mouse osteosarcoma allograft (CaOS18) and human osteosarcoma xenografts (B143, SJSA) treated with vehicle control or 20mg/kg sonidegib. All data are represented as mean + SEM of biological replicates. **P*<0.05, Mann-Whitney test. **b** Kaplan-Meier analysis of survival. **P* < 0.05, Log-rank (Mantel-Cox) test.

**Supplementary Fig. 12 Effect of *Shh* inactivation on mouse osteosarcoma growth *in vivo*. a** Western blot analysis of SHH and ACTIN expression in the of *p53Rb* KO mouse osteosarcoma cell line, D12M, following lenti-viral transduction of lenti-control or *Shh* gRNAs and single cell cloning. **b** SHH expression detected by immunohistochemistry in *Osx* *p53Rb* KO mouse osteosarcoma allografts (D12M) transduced with lenti-control or Shh gRNA. Scale bar = 200µm. **c** Tumor volume of *p53Rb* KO mouse osteosarcoma isogenic allografts (D12M) and treated with vehicle control or 20mg/kg sonidegib. n = 6, all data are represented as mean + SEM of biological replicates. **P*<0.05, ***P*<0.01, ****P*<0.001, Mann-Whitney test. **b** Kaplan-Meier analysis of survival. n = 6, **P* < 0.05.

**Supplementary Fig. 13 Effect of Smoothened inhibition on human and mouse osteosarcoma *in vivo.*** **a** GLI2, SHH, PCNA and cleaved Caspase-3 (c-CASP3) expression detected by immunohistochemistry in *Osx* *p53Rb* KO mouse osteosarcoma allografts (D12M) and human osteosarcoma xenografts (MG63, U2OS) treated with vehicle control or 20mg/kg sonidegib. Immunoperoxidase signal is shown in brown, counterstained with hematoxylin. Representative images are shown. Scale bar = 200µm. Quantification of GLI2, PCNA and c-CASP3 immunohistochemistry in D12M allograft tumors (**b**), and MG63 (**c**) and U2OS (**d**) xenograft tumors treated with vehicle control or 20mg/kg sonidegib (LDE225). *n* = 3 independent animals. All data are represented as mean + SEM of biological replicates. **P<0.05*, ***P* < 0.01, ****P<0.001*, Student’s unpaired t-test.

**Supplementary Fig. 14 Effect of Smoothened inhibition on human and mouse osteosarcoma *in vivo.*** Immunofluorescence colocalization of ARL13B in Osx *p53Rb* KO mouse osteosarcoma allografts (D12M) and human osteosarcoma xenografts (MG63, U2OS) treated with vehicle control or 20mg/kg sonidegib (LDE225). Scale bar = 15µm. Primary cilia frequency in D12M allograft tumors treated with vehicle control or 20mg/kg sonidegib (LDE225), and MG63 and U2OS xenograft tumors. *n* = 4 independent animals, mean + SEM.

**Supplementary Table 1. Genotyping primer sequences*.***

**Supplementary Table 2. gRNA and siRNA sequences*.***

**Supplementary Table 3. Quantitative RT-PCR primer sequences*.***

**Supplementary Table 4. Western blot antibodies.**

**Supplementary Table 5. Immunofluorescence antibodies*.***

**Supplementary Table 6. Sequencing primers*.***

**Supplementary Table 7. Immunohistochemistry antibodies*.***
