# Supplementary figures and images for: Ligand-dependent hedgehog signaling maintains an undifferentiated, malignant osteosarcoma phenotype
Source: Oncogene. 2023 Oct 16;42(47):3529–41. doi: 10.1038/s41388-023-02864-7 (PMC10656285; doi:10.1038/s41388-023-02864-7)

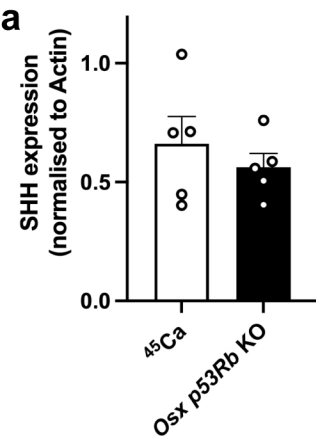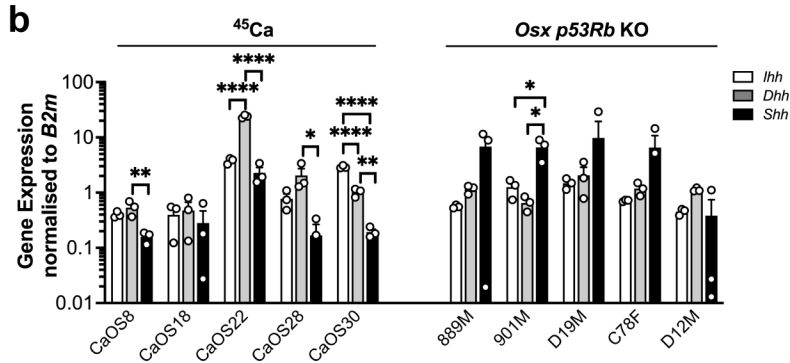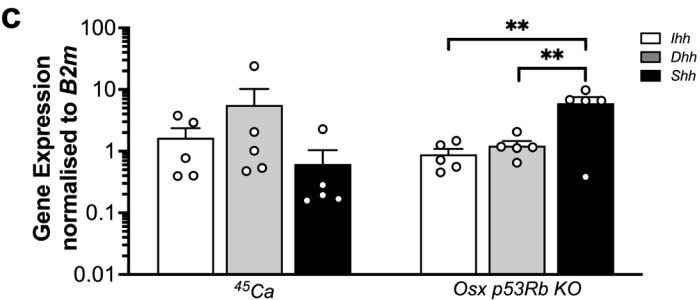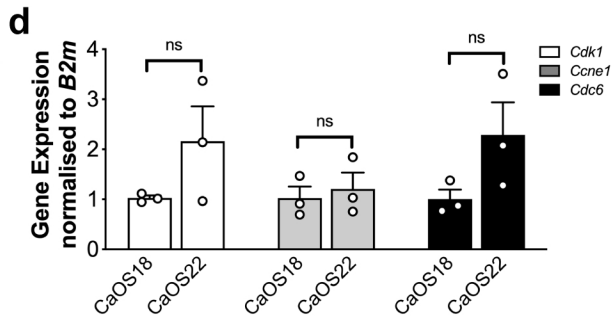

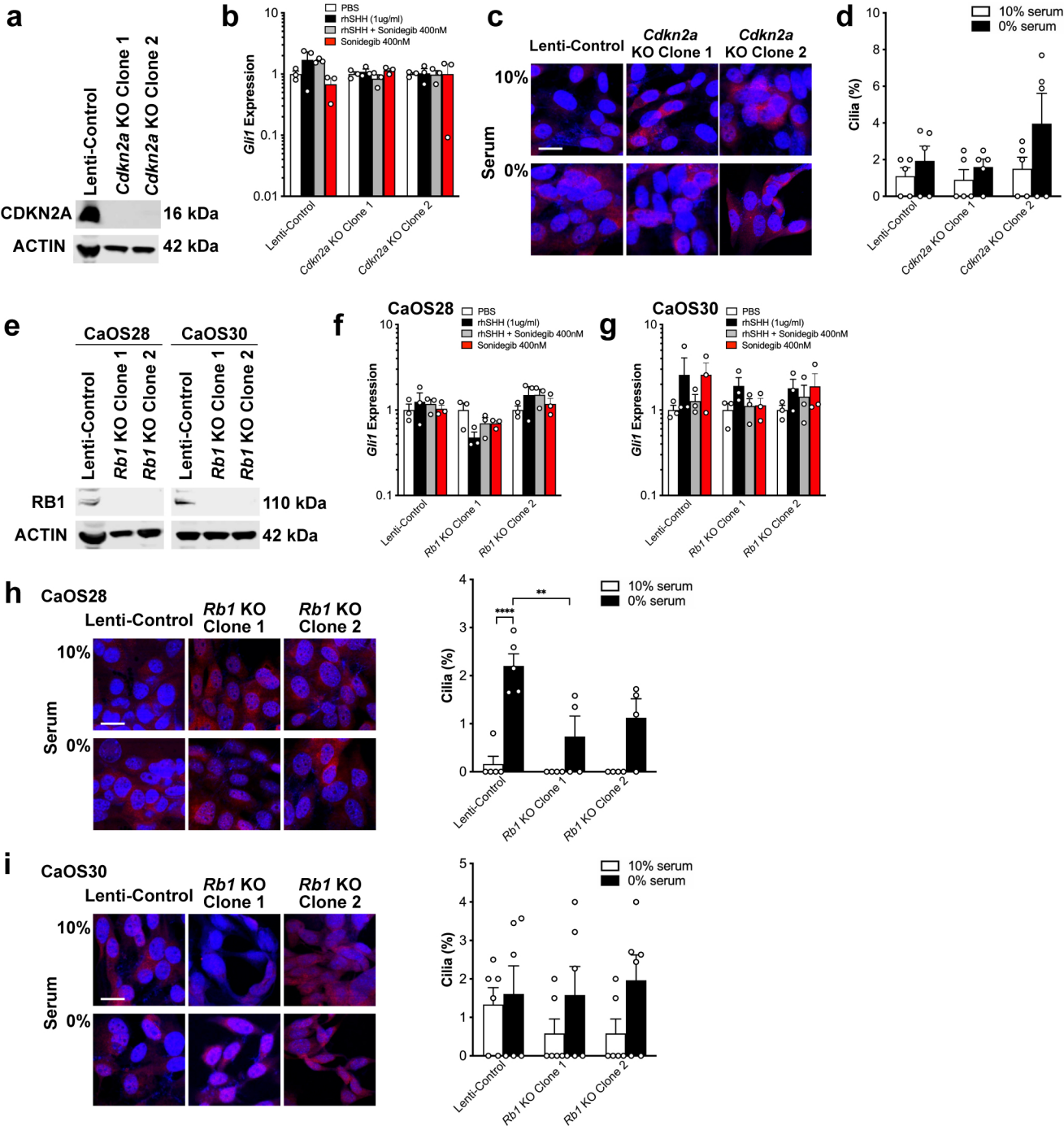

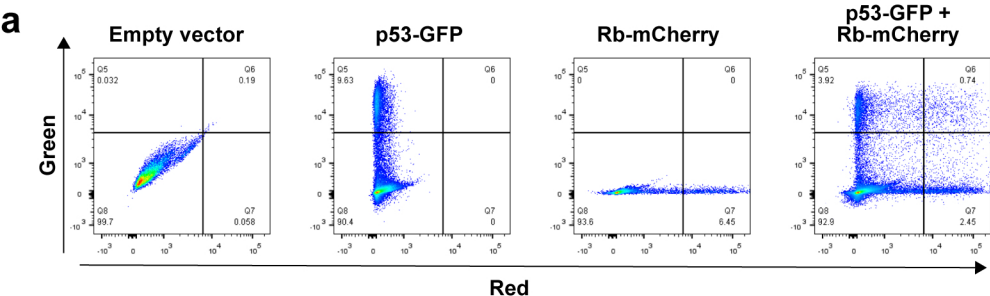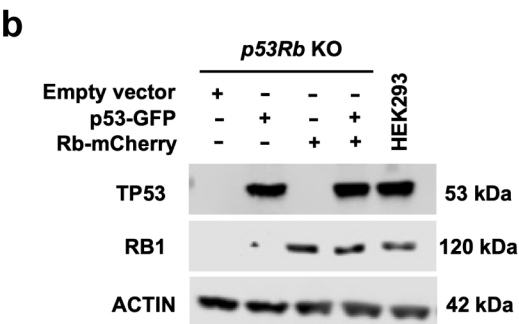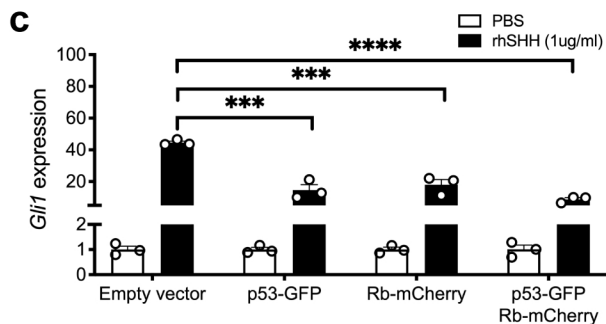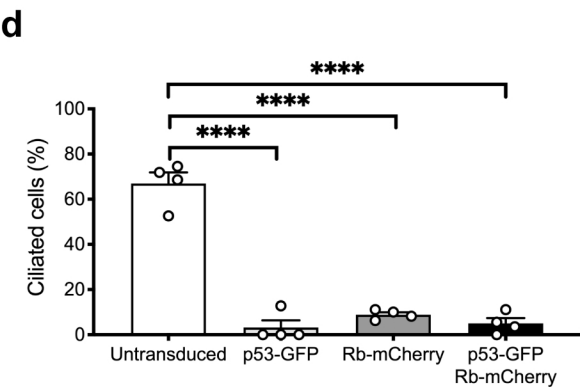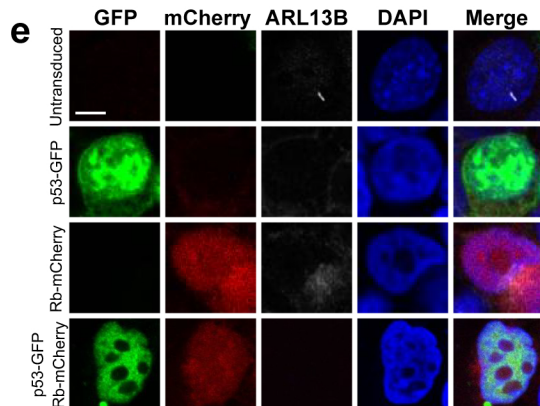

**a**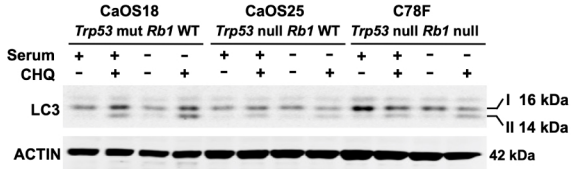**b**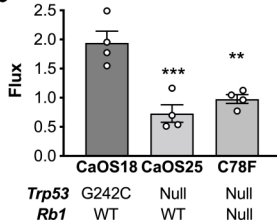

**a**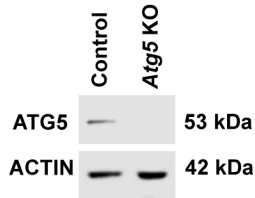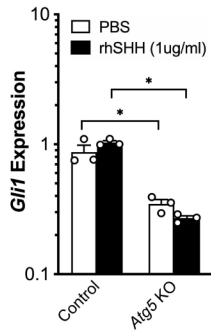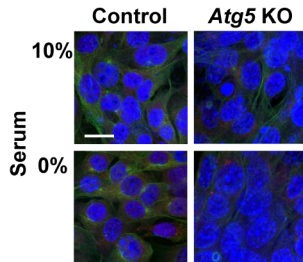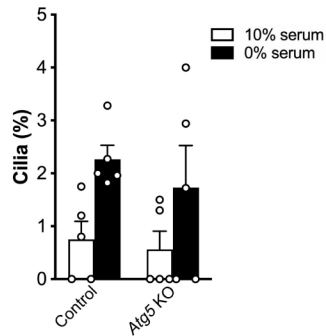**b**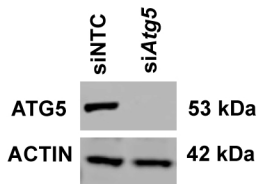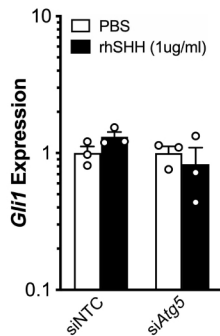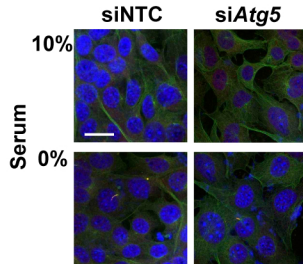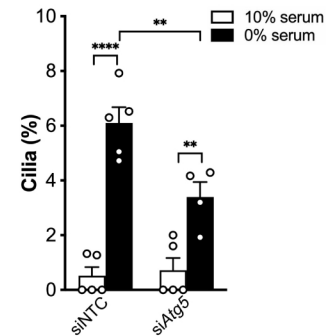

**a**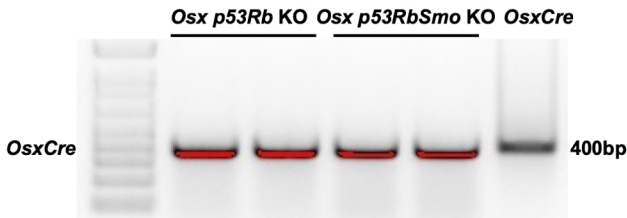**b**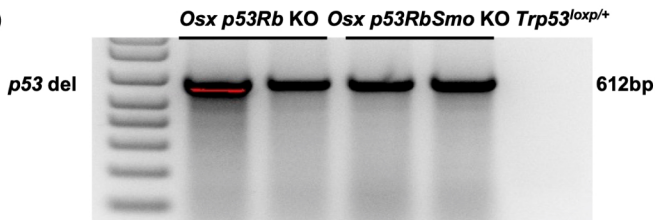**c**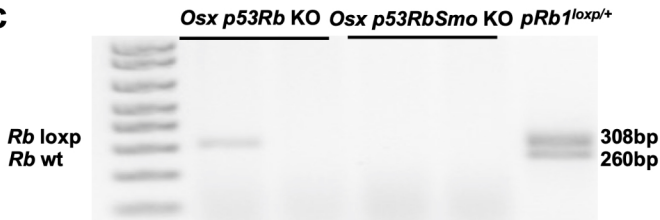**d**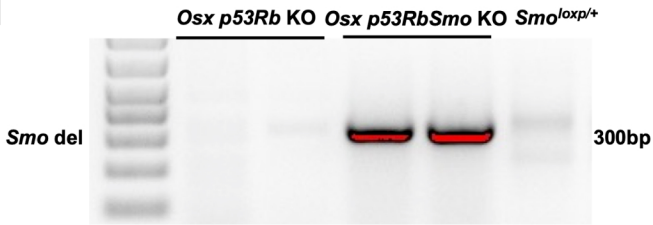

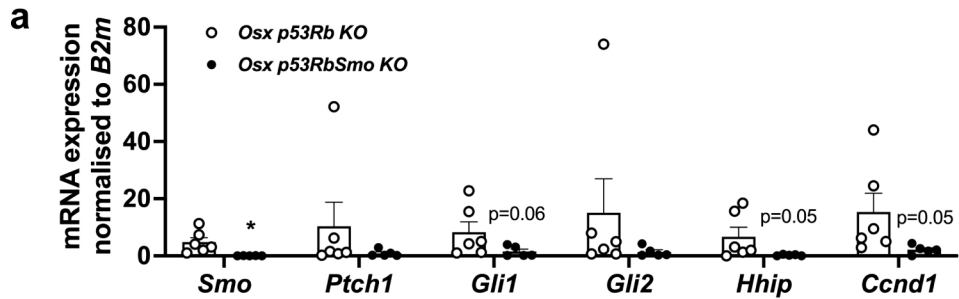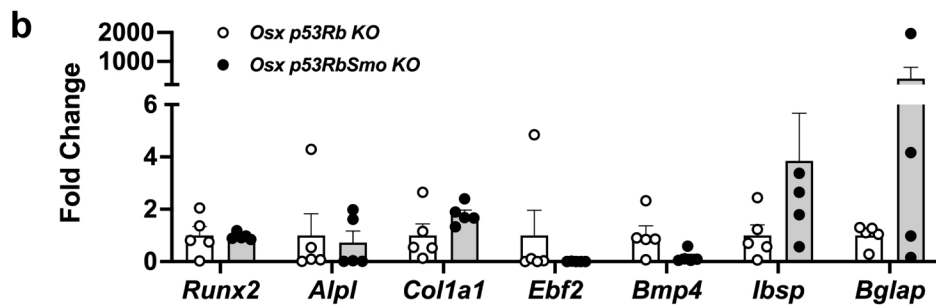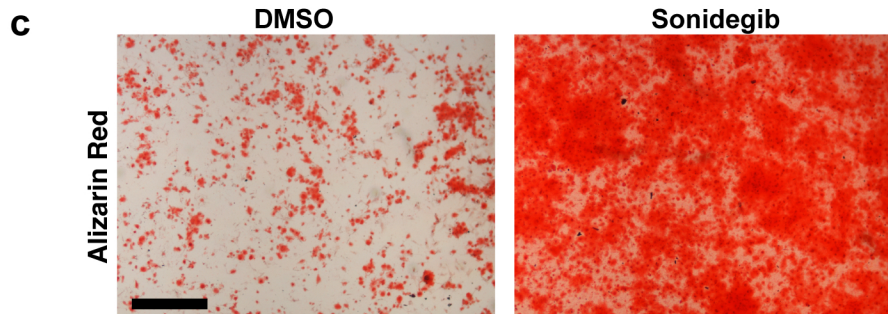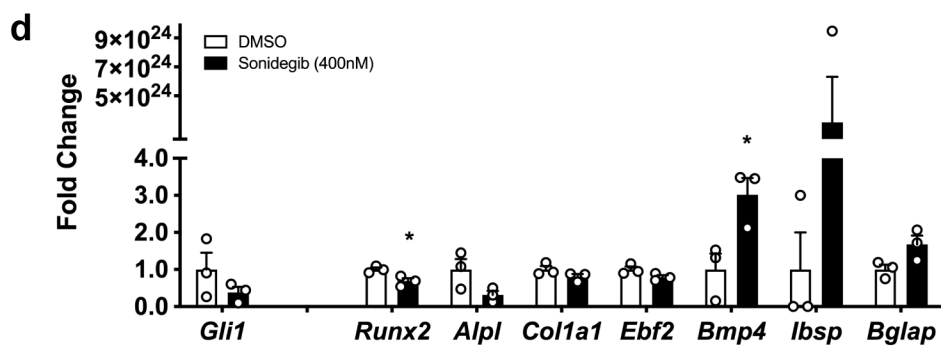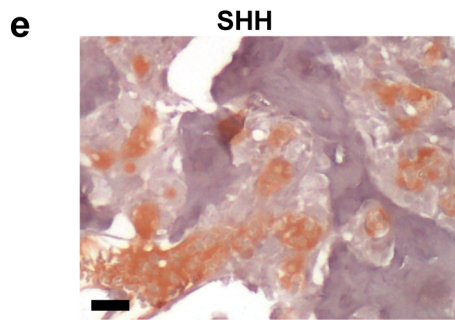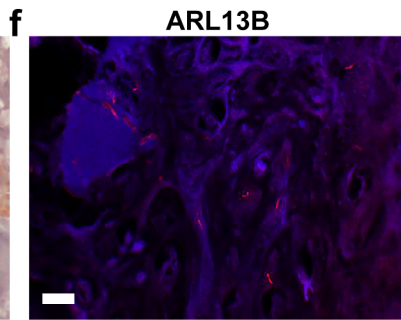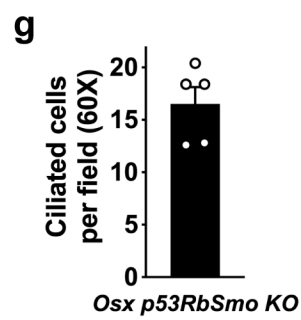

**a**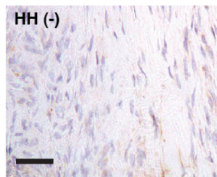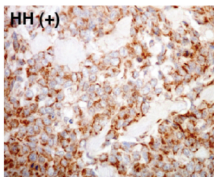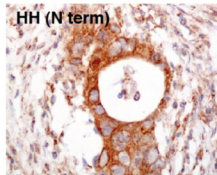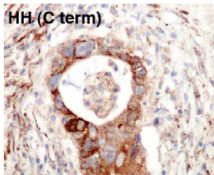**b**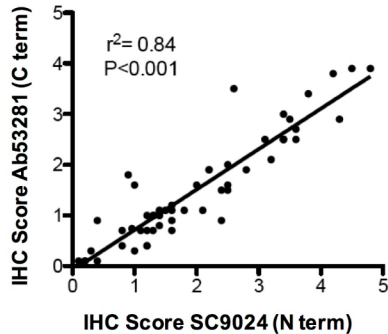**c**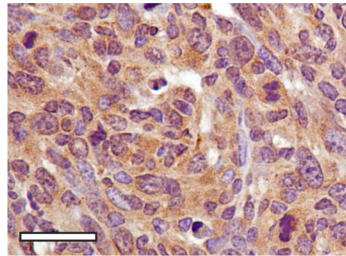

**a**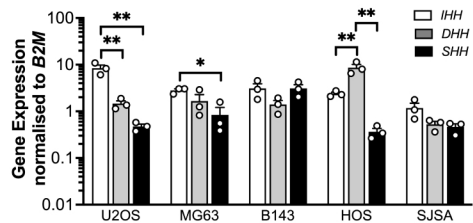**b**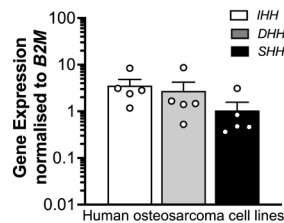**c**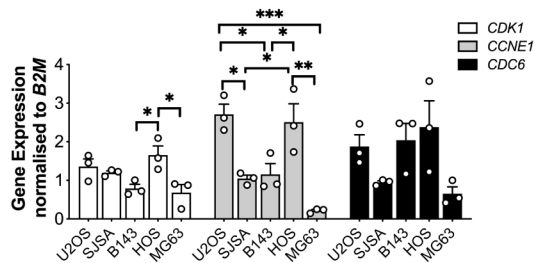**d**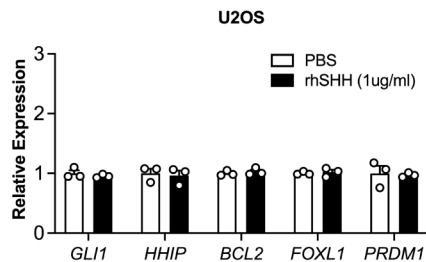**e**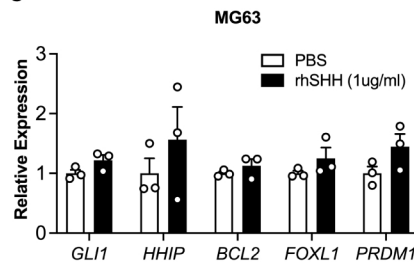**f**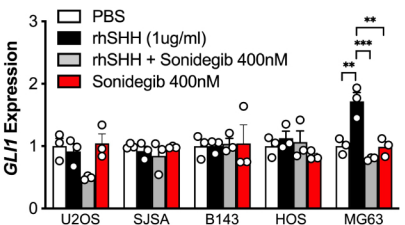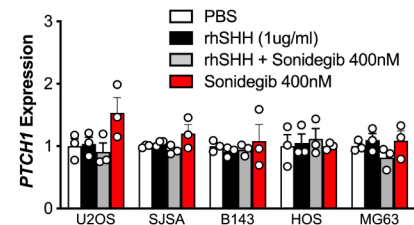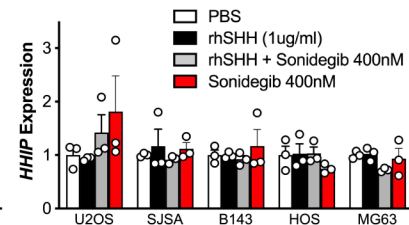**g**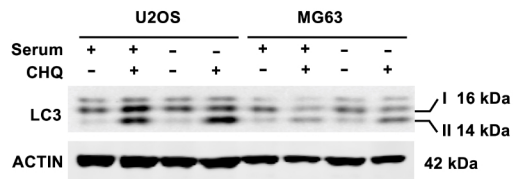**h**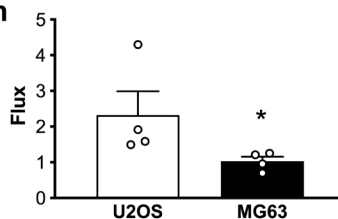

a

Control

*Osx* p53 KO*Osx* Rb KO*Osx* p53Rb KO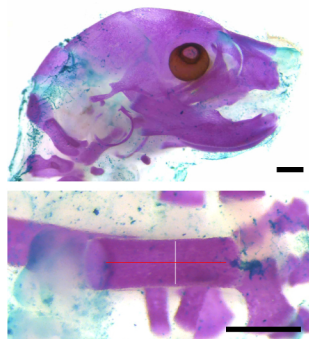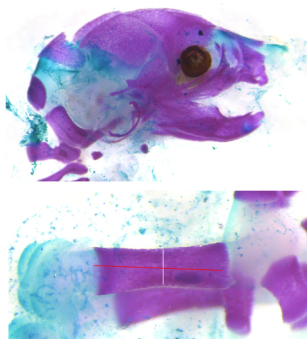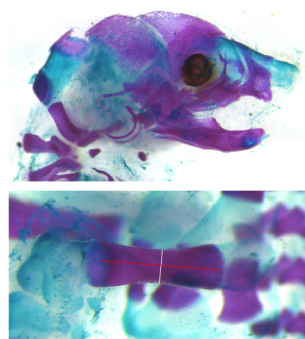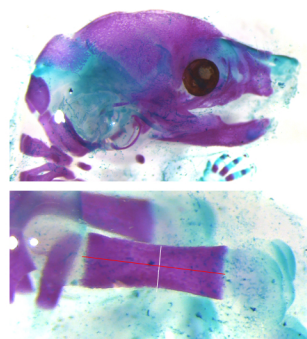

b

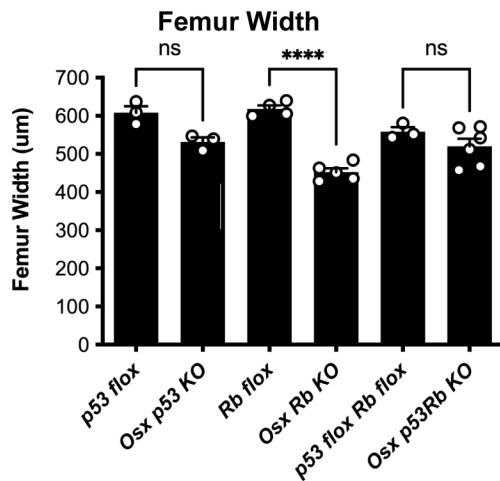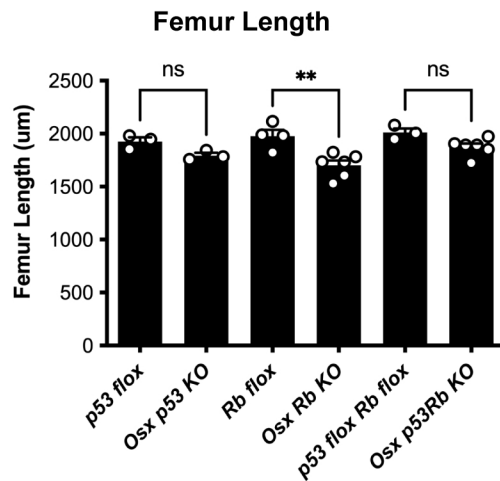

**a****CaOS18**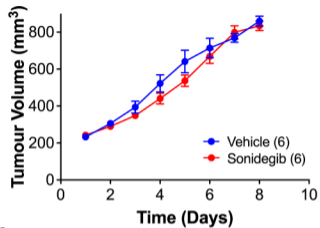**B143**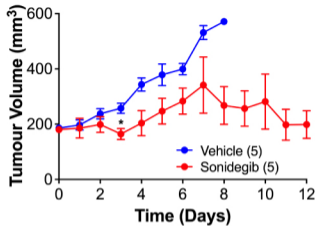**SJSA**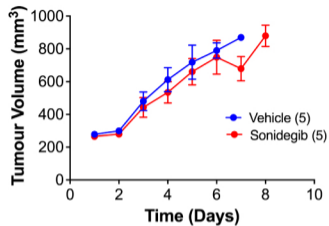**b**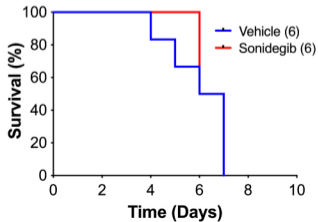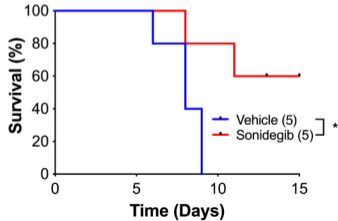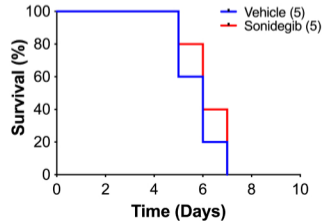

**a**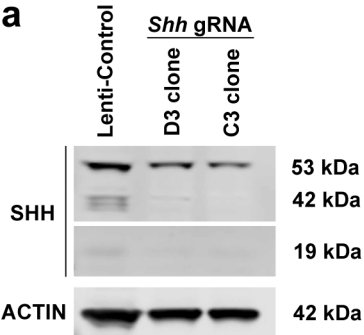**b**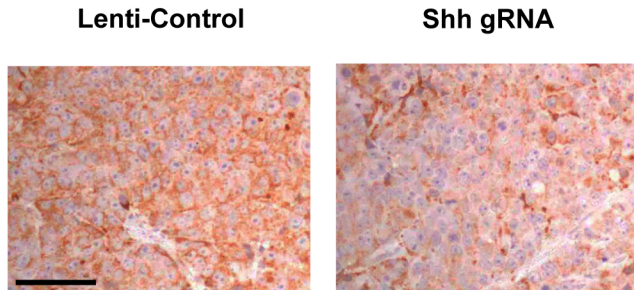**c**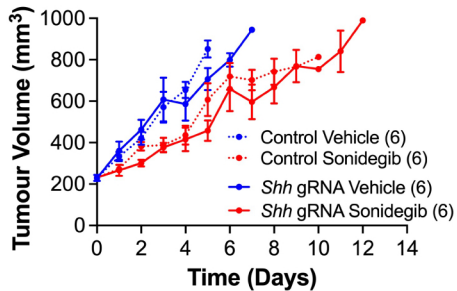**d**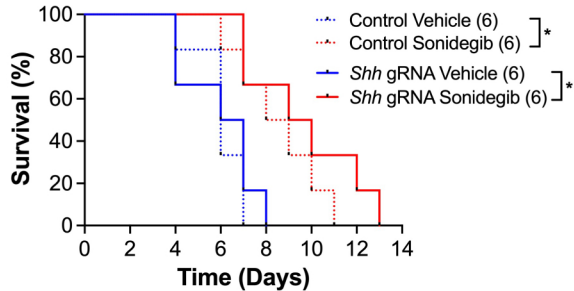

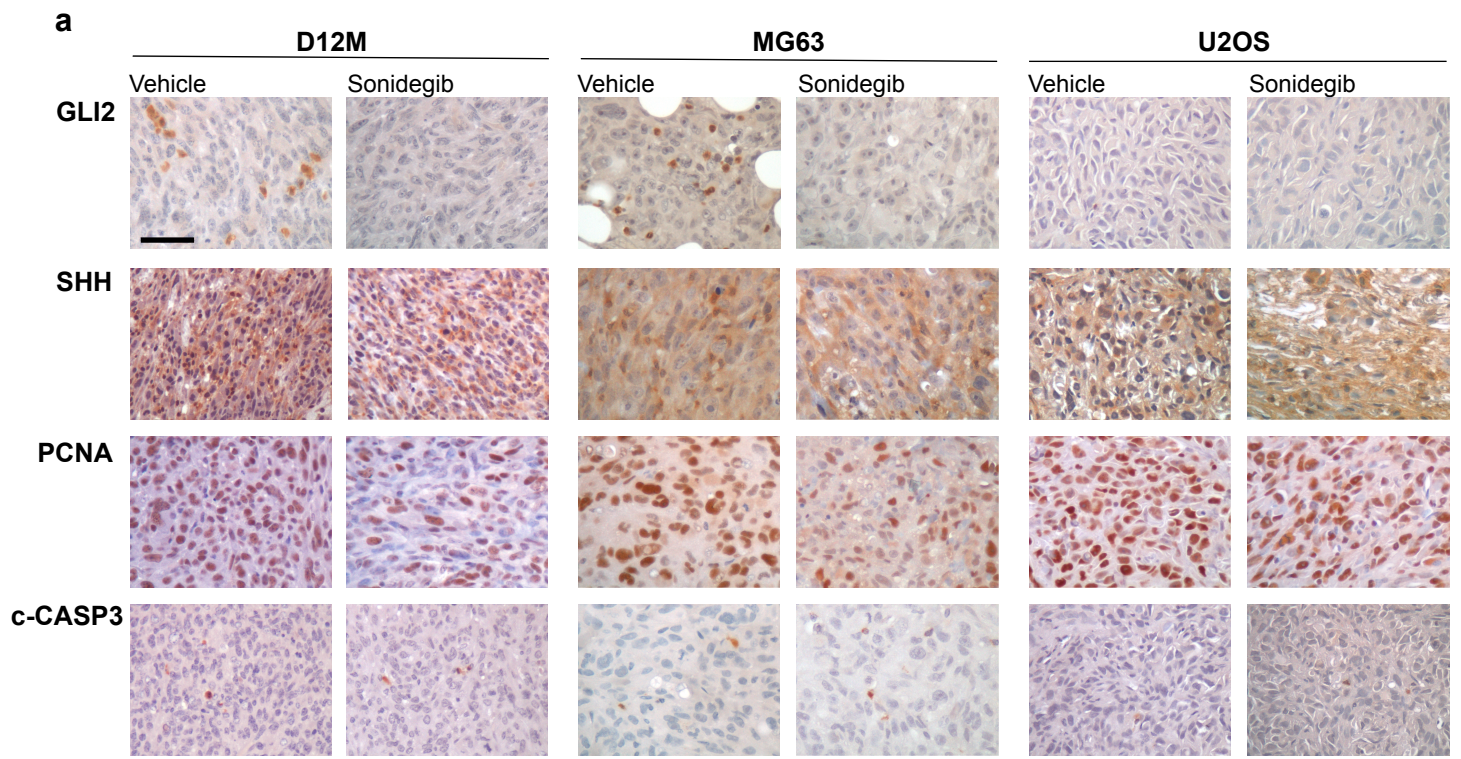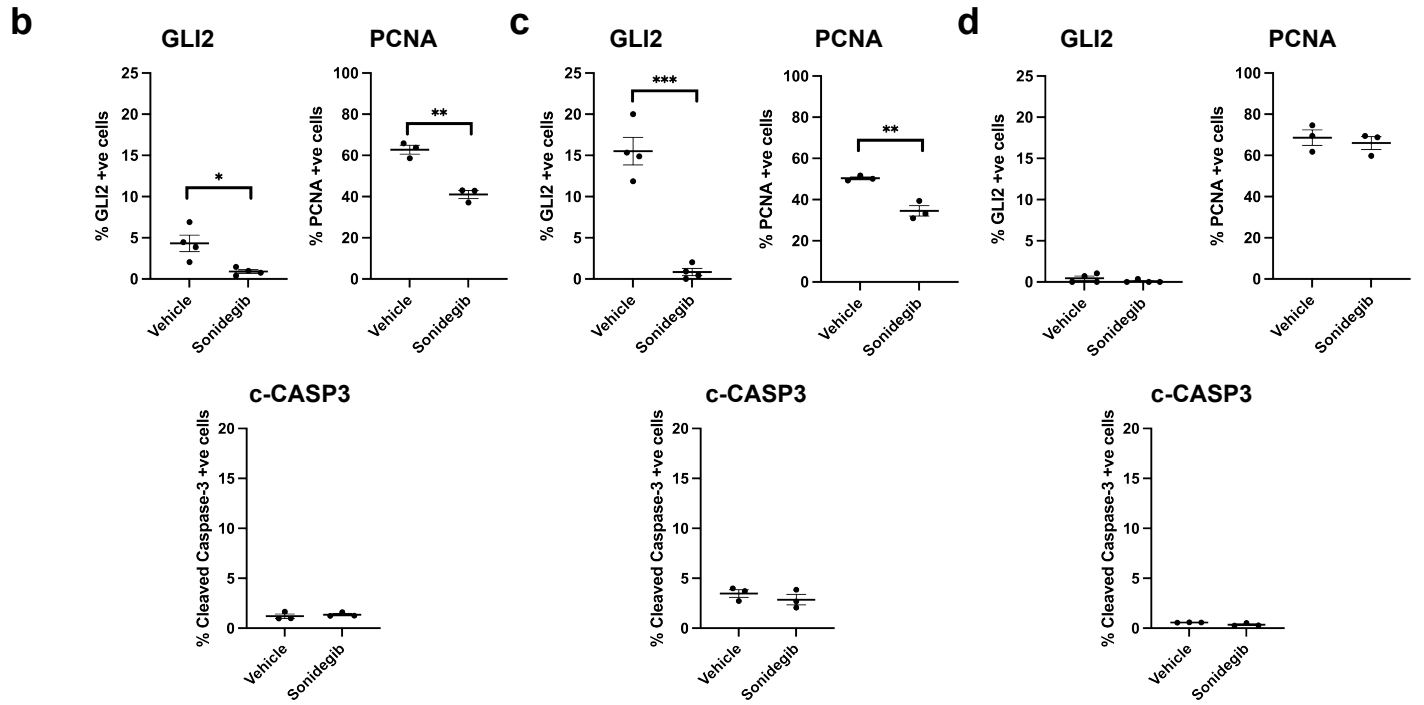

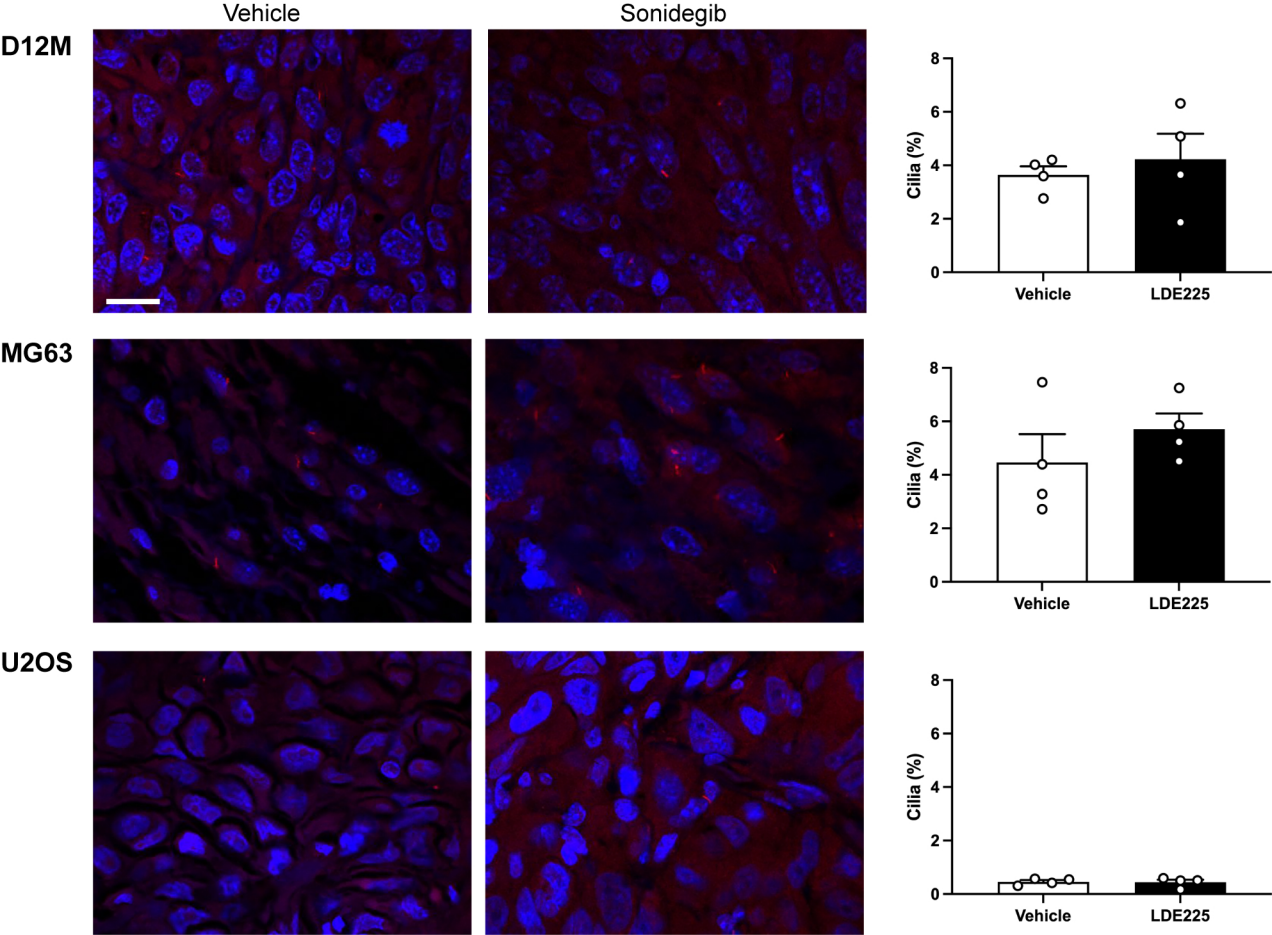

Supplement: Supplementary file 2 — Supplementary Figs. 1–14 [file 41388_2023_2864_MOESM2_ESM.pdf]
